# The Differential Warming Response of Britain’s Rivers (1982–2011)

**DOI:** 10.1371/journal.pone.0166247

**Published:** 2016-11-10

**Authors:** Art R. T. Jonkers, Kieran J. Sharkey

**Affiliations:** 1 Department of Mathematical Sciences, University of Liverpool, Liverpool, United Kingdom; 2 Institute for Geophysics, Westfälische Wilhelms Universität, Münster, Germany; Centro de Investigacion Cientifica y de Educacion Superior de Ensenada Division de Fisica Aplicada, MEXICO

## Abstract

River water temperature is a hydrological feature primarily controlled by topographical, meteorological, climatological, and anthropogenic factors. For Britain, the study of freshwater temperatures has focussed mainly on observations made in England and Wales; similar comprehensive data sets for Scotland are currently unavailable. Here we present a model for the whole of mainland Britain over three recent decades (1982–2011) that incorporates geographical extrapolation to Scotland. The model estimates daily mean freshwater temperature for every river segment and for any day in the studied period, based upon physico-geographical features, daily mean air and sea temperatures, and available freshwater temperature measurements. We also extrapolate the model temporally to predict future warming of Britain’s rivers given current observed trends. Our results highlight the spatial and temporal diversity of British freshwater temperatures and warming rates. Over the studied period, Britain’s rivers had a mean temperature of 9.84°C and experienced a mean warming of +0.22°C per decade, with lower rates for segments near lakes and in coastal regions. Model results indicate April as the fastest-warming month (+0.63°C per decade on average), and show that most rivers spend on average ever more days of the year at temperatures exceeding 10°C, a critical threshold for several fish pathogens. Our results also identify exceptional warming in parts of the Scottish Highlands (in April and September) and pervasive cooling episodes, in December throughout Britain and in July in the southwest of England (in Wales, Cornwall, Devon, and Dorset). This regional heterogeneity in rates of change has ramifications for current and future water quality, aquatic ecosystems, as well as for the spread of waterborne diseases.

## Introduction

The relevance of freshwater temperatures to hydrology, biology, and ecology has long been recognised [1,2]. Their variability on timescales from diel through seasonal and annual to multi-decadal is due to the complex interplay of natural processes and local conditions involving solar radiation (the dominant factor), topography, drainage basin, bathymetry, discharge flux dynamics, groundwater seepage, precipitation, and riparian and hill shading [3–8]. Additional human influences include landscape change (urbanisation, deforestation, agriculture, flow modifications), thermal pollution (e.g., power station cooling), and anthropogenic climate change [9–12].

Several large studies have identified ambient air temperature as the pre-eminent predictor for river temperature (e.g., in Alpine regions [13–15] and major U.S. rivers [16]). In Europe, an effect due to the North Atlantic Oscillation advecting heat and moisture across Europe has also been postulated [14–15, 17–18]. However, the impact of sea surface temperatures (SST) on freshwaters has received less attention.

Unlike previous studies that identified local and regional hydrological features in England and Wales based upon local measurements [2, 19], we wish to investigate freshwater thermal regimes throughout mainland Britain, including the whole of Scotland. Given the absence of comprehensive measurements from Scotland, we set out to construct a Britain-wide model that would relate local stream topography and recorded mean air and sea temperatures to the empirical record where and when available, so as to enable freshwater temperature to be estimated wherever and whenever direct measurements are absent in the studied region and period.

Our starting point for investigating thermal regimes in British freshwaters was a publicly-available, Europe-wide geographical information system (GIS) that contains extensive hydrographical and topographical information on river segments (small sections of rivers defined by this database), such as location, slope, elevation, nearby lakes, and distance to the sea. The underlying idea was to derive a numerical model that would relate these physico-geographical properties and local time-varying air and sea temperatures to temperature measurements made in specific river segments. By identifying adequate predictors that capture how freshwater temperature responds to changes in these factors, we can generate time series for any desired British river section for any period covered by these resources, even if no direct measurements of water temperature were made there at the time. This provides a uniform basis with which to analyse regional differences, in absolute terms, in observed differential warming rates, and in projections of future change. Thus our aims in this study were:

1. to derive an expression of daily mean freshwater temperature in terms of the most important topographical and time-dependent factors based upon river segment data, local air and sea temperature, and daily means of water temperature observations made in England and Wales over the period 1982–2011 (three full decades);
2. to reconstruct daily mean river water temperatures during this period for each river segment throughout Britain, including geographical extrapolation to Scotland, for which no published observations for the studied period are presently available to us; and
3. to quantify long-term warming trends per river segment, annually, seasonally, and monthly, so as to identify how and where river water temperatures have changed in the past and may do so in future.

The focus here is on mainland Britain, which has an oceanic (temperate maritime) climate; we therefore expect nearby sea temperature to be a relevant factor, particularly in coastal regions. Our area of interest includes the Orkneys, Shetlands, and Isle of Wight, but excludes Gibraltar, the Isle of Man, the Channel Islands, and Northern Ireland. For the targeted region, two high-quality data sets are publicly available that allow a high-resolution description of daily mean air and sea temperatures respectively. The oceanic record is available from 1982 up to the present day [20]; the dataset of inland air temperatures for the United Kingdom overlaps with the oceanic one for thirty years, from 1982 to 2011 inclusive. These three decades define the period of interest in this study.

In addition to daily mean air and sea temperatures, a large data set of observed freshwater temperatures is publicly available that contains incidental spot measurements and some sustained time series. This resource covers multiple decades of empirical observation at individual sites in England and Wales. However, these data do not extend to Scotland, and no such Scottish data archive covering the past few decades is currently available to us, nor does it seem likely (following extensive inquiries) that a Scottish resource of similar scope and magnitude for the same period will become publicly accessible in the near future. However, the Scottish land border is hydrologically and climatologically an artificial divide, straddled by two large river catchments, the Esk and the Tweed. This is why we consider the whole of mainland Britain as a single geographical entity.

The following two sections detail how the stated three aims of this study were achieved; the sections thereafter discuss the findings we were able to extract from the model-generated estimates. Full time series for each individual British river segment have been made available online for download [21].

## Materials and Methods

### Data Description

The four data sets listed in Table 1 provided the parameters and observations with which to establish a river water temperature modelling foundation that relates physico-geographical features, gridded local daily means of observed air temperature, and gridded nearby sea surface temperature to daily means of historical measurements of British freshwater temperatures. Each data set has been made freely available online by the agency responsible for compiling it.

**Table 1:** The four publicly available data sets used in this study.

Data Set (source)	Data Type	Number of Data points	Spatial coverage
ECRINS v.1.1 (EEA)	Physico-geographical	20,578 river segments	mainland Britain
UKCP09 (U.K. MetOffice), 1962–2011	Air temperature	113,420,691 daily means (1982–2011)	5 x 5 km U.K. Ordnance Survey grid [0–140 Easting, 0–250 Northing]
OISST v.02r00 (NOAA), 1982- (ongoing)	Sea Surface Temperature	17,936,100 daily means (1982–2011)	quarter-degree lat/lon grid [8W – 3 E, 49–62 N]
Surface Water Temperature Archive (U.K. EA/CCW), 1952–2008	Fresh water temperature	3,113,018 weighted daily means (1982–2008)	30,326 unique locations in England and Wales

The physico-geographical foundation of our modelling approach is a subset of the European Environment Agency’s “European Catchments and Rivers Network System,” or ECRINS database [22], comprising 20,578 river segments that together form 1,482 separate catchments in mainland Britain. Each catchment consists of at least one segment, and each segment is defined by an upstream (“headwater”) and a downstream node (“terminus”). British segment lengths range from 100m to 46 km; their mean length is 2,575m.

From the various ECRINS resources at our disposal, we built a database of British segments that recorded the river slope (“pente”), its elevation (in meters) and the coordinates of headwater and terminus (latitude and longitude, and Ordnance Survey grid easting and northing). We subsequently defined a third point midway, taking the mean of the OS coordinates, which provided the reference for associating each segment with historical measurement sites and grid cell centroids from the other databases (see below). We defined each catchment by aggregating each set of connected segments; that is, ones that share a node with their immediate neighbour(s). Each composite chain may have multiple starting points that transport water from drainage sub-basins that confluence at junction nodes, ending up at a single, final node where the chain meets the sea.

The second main input of our models is daily mean air temperature (AT) collected by the U.K.’s meteorological office (hereafter: “MetOffice”). It is stored in the gridded observation data sets known collectively as UKCP09 [23]. These span the period 1962–2011 and cover the United Kingdom at 5 × 5 km resolution, yielding a rectangular grid of 250 rows by 140 columns. Each ECRINS river segment midpoint was associated with the nearest UKCP09 grid cell’s midpoint (based upon Euclidean distance), and thereby, with that cell’s time series of daily mean air temperature. The third main data resource contains Sea Surface Temperatures (SST), provided on a daily basis by the U.S. National Oceanic & Atmospheric Administration (NOAA) at a grid resolution of one quarter-degree [20, 24]. We treated this oceanic temperature grid in the same fashion, associating each river segment with the nearest sea grid cell by comparing their centroid’s Euclidean distances to the segment midpoint.

The combination of ECRINS physico-geographical features and SST and UKCP09 data sets of daily mean temperatures provides a robust framework of interpretation. Each one is independently collected by experts and subjected to extensive quality control prior to publication by their respective agencies (EEA, NOAA, and the U.K.’s MetOffice). The last data set used here is less satisfactory in this respect. The Surface Water Temperature Archive (SWTA) has dense, but irregular spatial coverage from the early 1980s onward [25–27, 2] that comprises raw data of incidental spot measurements and some sustained time series. These archives concern historical measurements of British river and lake water temperatures collected since the 1950s in England and Wales. The SWTA was published in 2012 by the U.K.’s Environment Agency in collaboration with the Countryside Council for Wales. From this resource we derived 3,113,018 sample size-weighted daily means for the modelling period of 1982–2011, and associated each sampled location with the nearest MetOffice grid cell centroid, the nearest SST grid cell centroid, and the nearest ECRINS river segment. Additional information about these four data sets is provided in Text A in S1 File.

We classified all ECRINS segments into one of three classes: coastal (near to the coast), lacustrine (near a lake), or riverine (the remainder), as we expect that large bodies of water (seas and lakes) have a significant, distinct effect on nearby freshwater temperatures, requiring separate treatment. By definition, the final segment of each catchment chain was placed in the coastal class. Using the segments’ distance to the sea, we assigned the same class to those whose midpoint was located less than 10 km from the coast, yielding 1,845 coastal (C) segments in total. Neither criterion on its own is sufficient; the midpoint of long final segments may be further away, and intermediate segments may flow parallel to the coastline, while remaining in close proximity to the sea.

In similar fashion, we used the ECRINS data set of lake centroids to compute each segment’s Euclidean distance to the nearest lake (in meters), classifying 753 non-coastal segments as lacustrine (L) for being within 1 km of any lake centroid. The remaining 17,980 segments were then assigned class riverine (R). This three-way classification yielded three separate data sets and three separate sets of modelling parameters; thus our overall model for Britain’s freshwaters consists of three sub models, one for each segment class.

In the absence of Scottish observations of freshwater temperatures, the physico-geographical modelling parameters (in particular, steep-sloped, high-elevation river segments relatively close to the coast as found in parts of Wales and Cumbria) provided proxies for similar segments in the Scottish Highlands. We therefore need to address to what extent it is reasonable to extrapolate the derived relationships between model parameters and river water temperatures as observed in England and Wales, which extend to about 56 degrees north latitude, to Scotland. Partitioning the ECRINS data at that latitude yields 12,453 segments to the south of 56°N and 8,125 to the north of this divide. Regarding elevation, 425 southern segments exceed 300m (maximum 545m), against 2,186 in the north. Of the northern segments, 389 (less than 4.8%) exceed the maximum height of southern topography. Thus the majority of segment elevations found in Scotland are also present among the English and Welsh segments.

In terms of river segment slope (which Pearson-correlates moderately with elevation, at +0.58), a mere seven northern segments (0.09%) exceed the maximum slope (36.3) observed in the south. Distance from the sea is comparable in all three countries as well, and we expect much of the remaining differences in topography-related effects and other influences to be reflected in the locally recorded air and sea temperatures, for which daily high-resolution grid values *are* available over the studied period for the whole of Scotland. Moreover, it is these latter, time-variant model inputs that were found to dominate the model response (see next section).

The three time-dependent data sets used in this study are different in several respects. Spatially, the SWTA observations are made at point locations, whereas the ECRINS segment can be considered to be lines (vectors) crossing a region. Secondly, the grid resolution over land (5 x 5 km) is almost an order of magnitude higher than over sea, but because the river segments are on average of similar size as air temperature grid cells, and are by definition found only inland, we did not downscale the AT grid to match the lower SST resolution. Thirdly, as stated, the SWTA measurements do not include Scotland, whereas the other three data sets do.

Another difference between the data sets concerns the temperature values themselves. We obtained SST as daily means, whereas the MetOffice’s UKCP09 set offers daily minimum, mean, and maximum air temperature, and the SWTA contains raw observations ranging from isolated single values to over a hundred measurements per day for a single site. Wherever multiple freshwater observations per day are available at a location, their mean, minimum, or maximum could in theory be extracted. However, in the studied period, such days make up only 10.8% of the SWTA data. As spot measurements are more likely to be nearer the daily mean than to either extremum, and minimum and maximum are the least robust descriptive statistics available, we followed the most consistent approach in using daily means from all data sets (see also Text A in S1 File).

### Modelling Methodology

Reducing a large, multivariate, time-dependent, empirical dataset to a handful of parameters implies confronting the trade-off between model complexity and misfit with the observations, for which numerous strategies have been devised. The more parameters are available to capture data features, the more complicated the model becomes, while gaining a higher percentage of total data variance explained (goodness-of-fit, R^2^). For the current purpose of deriving expressions that are generally applicable throughout mainland Britain, we chose a statistical criterion to determine how complex the model(s) should be. We started by storing the previous fifteen days of local air and sea data for each daily mean of historical measurements, together with the fixed-value properties of the associated river segment such as coordinates, slope, and elevation. We then ran large suites of sample size-weighted least-squares test models in which parameters were systematically included and excluded in successive permutations, while the cut-off for the number of days prior to the day of observation was moved from zero to fifteen days. This was done separately for air and sea temperatures.

We then identified those models with the highest R^2^ values while rejecting all models for which any parameter had either a zero coefficient, or its *p*-value exceeded 0.01. A detailed description of this method with several examples is provided in Text B in S1 File. We note that goodness-of-fit by itself is an insufficient criterion for model selection, as it proved feasible to keep marginally increasing this metric by adding ever more parameters (overfitting), but at the expense of causing unacceptably high *p*-values to appear (that is, a one-sided confidence interval above 99% was imposed; one-sided because the *p*-value expresses the likelihood of results being due to chance alone, so a single bound separates the acceptable from the unacceptable region of the *p*-value distribution, defining the acceptance level).

Given the threefold division of segments, the final parameter ensemble is different per class (Table 2). For example, unsurprisingly, sea distance is close to zero for all coastal sites, but was found to make a statistically significant contribution to the model further inland. Likewise, river slope (deemed a proxy for river flux rate in hilly terrain) is near-zero at the coast and along lakes, and thus disregarded there. Other parameters were rejected because their *p*-value exceeded the imposed confidence limit. For example, northing and easting were found to significantly affect riverine segments, but not coastal or lacustrine ones. Other ECRINS-related candidate variables (river segment azimuth, downstream distance to river mouth, drainage basin area, Strahler confluence level) did not provide significant additional information for any segment class, and were thus also excluded.

**Table 2:** Segment class-specific model parameters for which coefficients are derived.

Variable	Description	Coastal	Lacustrine	Riverine
Constant	Intercept of the modelled river water temperature, in °C	Used	Used	Used
Northing	MetOffice UKCP09 grid row (1–250); one cell is 5 x 5 km (British Ordnance Survey grid)	Not Used	Not Used	Used
Easting	MetOffice UKCP09 grid column (1–140); one cell is 5 x 5 km (British Ordnance Survey grid)	Not Used	Not Used	Used
Elevation	ECRINS (river segment) database field, in meters (mean of start and end node height)	Used	Used	Used
Pente	ECRINS (river segment) database field, inverse slope ratio (larger is steeper)	Not Used	Not Used	Used
SeaDistance	Distance in km from segment midpoint to nearest NOAA SST grid cell centroid	Not Used	Used	Used
SeaTemp#	Mean SST in °C (NOAA), # days prior to prediction’s date at nearest sea grid cell	Days 0–1	Days 0–1	Day 0
AirTemp#	Mean air temperature in °C (UKCP09, MetOffice), # days prior to prediction’s date	Days 0–5	Days 0–8	Days 0–7

We assume that the relative differences in heat capacity of large water bodies (lakes and seas) as opposed to fast-flowing, comparatively shallow streams and rivers underlie the different time windows we derived in considering the effects of past air and sea temperatures: for coasts, six days of mean air temperature (AT) and two days of mean SST; for lakes, nine days of AT and two days of SST; and for rivers, eight days of AT but only the prediction day’s SST (Table 2). In all cases, these sampling windows include the day of the prediction itself. These periods were derived by running test models that explored *all* possible permutations of parameter ensembles, and identifying at which point additional parameters became statistically insignificant in terms of *p*-value or contribution to the model.

To test model performance further, we also partitioned the observational data temporally, and performed initial fits for data observed during the central two decades of the time window (1987–2006). All fits were class-specific, yielding three sub models per compound model. That is, one model was derived for all coastal segments, one for all lake-associated segments, and one for all river segments. Subsequently we computed normalised residuals (in-sample testing) and quantified the percentage of data variance accounted for by the model (goodness-of-fit). We then performed out-of-sample tests, comparing the model predictions with the ca. 470 thousand observations made outside the two central decades. No statistically significant differences were found between the hindcasting and forecasting accuracy of the out-of-sample predictions, or with the in-sample predictions covering the central two decades (see Figures B-C in S1 File).

The final model, based upon the full data set (1982–2011) is defined by the coefficients listed in Table 3. To derive an actual water temperature estimate from this model, one would 1) identify the segment class to fit, and select the appropriate column, 2) collect the required parameter values for the non-zero coefficients, and 3) add to the Constant term the sum of each coefficient multiplied by its associated parameter value (parameter units are listed in Table 2). The final paragraphs of Text B in S1 File contain a step-by-step example of how the model derives a freshwater temperature from given parameter inputs. Generated full time series (1982–2011) of model-estimated daily mean temperatures for each British river segment are freely available online [21].

**Table 3:** Final model coefficients (rows) for mainland Britain (1982–2011), per river segment class (columns).

	Coastal	Lacustrine	Riverine
Constant	0.5517	0.3041	0.5714
Northing	0	0	1.10617e-3
Easting	0	0	6.5541e-3
Elevation	2.8988e-2	1.9857e-3	-8.8737e-4
Pente	0	0	-1.0802e-1
SeaDistance	0	-1.2576e-2	6.6380e-3
SeaTemp0	2.0697e-1	2.6374e-1	1.3722e-1
SeaTemp1	2.7253e-1	2.8025e-1	0
AirTemp0	1.6347e-1	1.0314e-1	3.5338e-1
AirTemp1	7.6029e-2	6.5541e-2	1.3986e-1
AirTemp2	6.2363e-2	6.5078e-2	6.9966e-2
AirTemp3	6.2431e-2	4.9051e-2	5.6135e-2
AirTemp4	4.2267e-2	5.1148e-2	3.8138e-2
AirTemp5	1.3736e-1	4.6577e-2	3.4629e-2
AirTemp6	0	4.6846e-2	2.2768e-2
AirTemp7	0	2.6406e-2	6.3984e-2
AirTemp8	0	1.0143e-1	0

Note: a zero coefficient implies that this parameter is not used in the model estimate for the associated class. Parameter units are listed in Table 2.

The extent to which the final model fits the observations concerns the interrelated aspects of outlier rejection and model residuals (observed minus estimated value). Regarding the former, given the absence of extensive quality controls on the SWTA data, it was unsurprising to find these records to be of variable quality, with some physically implausible values likely due to uncorrected typographical errors. We deal with this issue in a purely statistical way. An initial model fit of the raw observations provided the basis for computing standardised residuals of the observations, which in turn allowed us to exclude any data whose absolute normalised distance from the mean exceeded some maximum-acceptable multiple of the standard deviation. In Table A in S1 File, we compare model coefficients, goodness-of-fit, and the number of rejected outliers for two different outlier rejection schemes against those for the raw data fit. The first of these schemes rejects outliers of the initial fit immediately at +/-2σ (sigma = standard deviation), whereas the second scheme achieved the same in a multi-step process that we prefer, rejecting first at +/-10σ, then at 5, 3, and 2σ, while recomputing a new model with the remaining data after each outlier-removal step. The advantage of this more gradual approach is that it allows the fit to get rid of differently-scaled sources of error and noise that may skew initial attempts in different directions. The total proportion of removed outliers consequently increases from about 5% for single-step rejection to about 8.5% for multi-step rejection. All results presented here are based upon the multi-step technique.

The iterative nature of outlier rejection at successively lower standard deviations is akin to the mathematical technique of simulated annealing, in that it allows the model to explore parameter space before settling upon a final optimum. In the presence of extreme outliers, this approach is more likely to identify the best global fit than when accepting the initially-identified outliers as the final word. However, its application does not imply that the freshwater inputs are all of poor quality or inherently biased; their larger variability may also reflect localised hydrological conditions such as groundwater contributions, evaporation rates, unusual sampling regimes, or other local factors that our model does not capture. Recent studies [2, 19] have focussed on the distinctions between regional data subsets with particular characteristics. By contrast, our aim was to derive the simplest robust parametrisation that would hold for all areas and sampling regimes for the entire SWTA data set (after reduction to daily means, and weighted by the number of observations taken that day), without predefining regional subsets with specific properties. We stress that entire sites were never removed from the record, only individual means for a particular day, and on purely statistical grounds; at 10σ, mostly transcription errors were likely to be caught, whereas at 2σ, the model likely removed some local spatial heterogeneities as explored in other studies. But given that a) removed outliers constitute less than 1/10th of the total data, b) the resulting model explains over 94% of all observed variability in the remaining ~2.8 million daily means (see below); c) mean model residuals are close to zero; d) derived warming rates broadly agree with previous studies, both for the region [2] and globally [28], we would argue that the model’s inability of predicting every local measurement exactly is a fair trade-off for achieving our stated aims.

Adjusted R^2^ values for the raw, unfiltered data were 90.3%, 85.8%, and 65.1% for coastal, lacustrine and riverine segments respectively. After the multi-step removal of outliers, these values improve to 94.6%, 93.9%, and 94.7% respectively. We believe that the river segment class increased most because it contains a much broader topographical variety of environments than the other two classes. Outlier removal in the river class is thus more likely to remove more observations that are farther from the mean than in the other two cases, and the end result will therefore have a more reduced spread with respect to the raw fit, causing a larger proportion of data variance to be captured by the model thereafter, yielding a greater improvement in R^2^. Mean data residuals likewise improved, from ca. -0.6°C for the raw data to close to zero after outlier rejection, with residual standard deviation below two degrees centigrade in all cases (less than one degree for coastal segments), and yielding a 95%-confidence interval of +/- 3.17°C overall (Table 4).

**Table 4:** Descriptive statistics of final model in-sample residuals, in °C.

Final Model	Observations	Mean	StDev	Variance	95%-conf. range
- coastal	13,627	0.000	0.930	0.864	+/- 1.86°C
- lacustrine	37,138	0.065	1.971	3.887	+/- 3.94°C
- riverine	2,763,342	-0.018	1.582	2.504	+/- 3.11°C
All segments	2,814,107	-0.017	1.586	2.515	+/- 3.17°C

Finally, we tested for, but did not find, significant spatial or temporal bias in the model residuals, nor with respect to any individual modelling parameter. Given that all parameter *p*-values (the likelihood of their contribution being due to chance alone) are effectively zero (below 5.0e-4), we conclude that the chosen parameter ensembles do not overfit the data, that is, each included parameter does statistically contribute significantly to the models’ overall goodness-of-fit.

Time series per segment were constructed by obtaining the relevant parameters and applying them with the linear model. Elevation of a segment was determined by taking the mean elevation between the upstream and downstream node. Air and sea temperature data were retrieved from the data sets for the moving window of prior days for each of the 10,948 days comprising the years 1982–2011. Note that the first nine days of 1982 are lacking, as the SST satellite record is available from Jan 1^st^ 1982 onward, and up to nine prior days are required to reconstruct the first model estimate. Thus for consistency, all time series start on 10 Jan 1982. We also computed per year the mean temperature per river segment for each month, season, and full year, and derived the mean rate of positive or negative warming per segment per case, by computing the slopes of least-squares fitted lines through these annual points (thirty points per fit, 20,578 fits per case). These form the basis for the results presented in the next section, and for segment-specific projections of future warming (details in Text B in S1 File).

## Results

**Fig 1:**
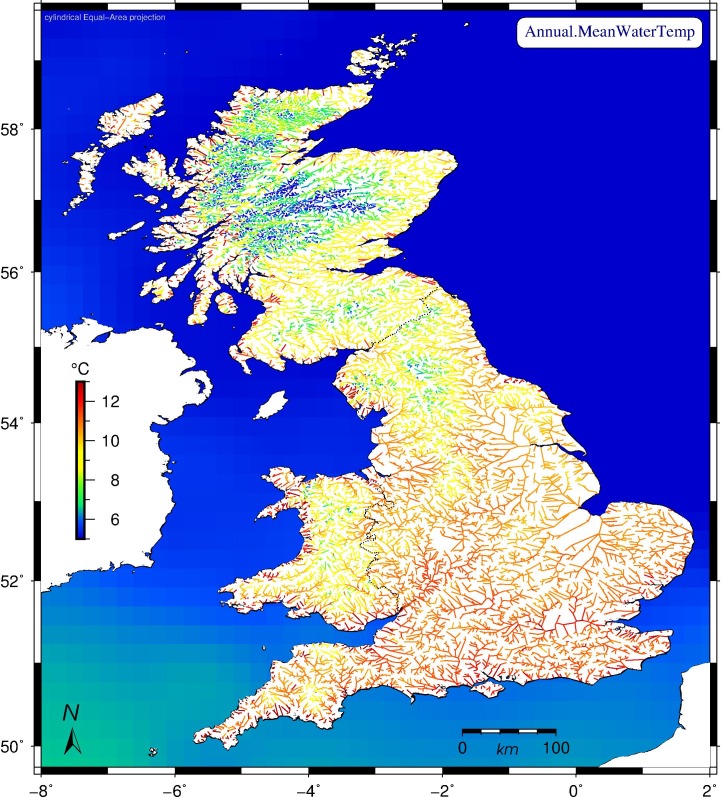
Modelled mean British water temperatures per river segment over the period 1982–2011. Freshwaters are on average warmer in England than in Scotland and Wales. Sea surface temperatures (SST, same colour scale) plotted per quarter-degree were derived directly from NOAA data sets of satellite measurements. Cylindrical equal-area projection. Scottish model results represent an extrapolation of English and Welsh data.

We separately explored model parametrisations for the 17,980 riverine, 753 lacustrine, and 1,845 coastal segments. We found that SST at the nearest coast could by itself explain 44.4% of total variance in observed riverine temperatures, 79% for lake-associated segments, and 82.1% close to the coast. Thus SST is clearly a significant contributing factor to the model. But a much improved fit was achieved by adding a class-specific time window of air temperatures on prior days, plus local river slope, elevation, and, for inland segments, their MetOffice grid coordinates and distance to the sea. The final model generated daily temperature estimates per segment for the period 1982–2011, from which local mean temperatures and warming rates were derived per annum, season, and month. Fig 1 displays the derived mean freshwater temperature for each segment over all three decades (midpoint: 1997), in order to provide a geographical baseline. Large parts of England’s southeast, as well as most coastal regions throughout Britain stand out as warmest, whereas the Scottish Highlands remained the coolest. We note furthermore the warm coastal segments, especially along Britain’s western shores.

**Fig 2:**
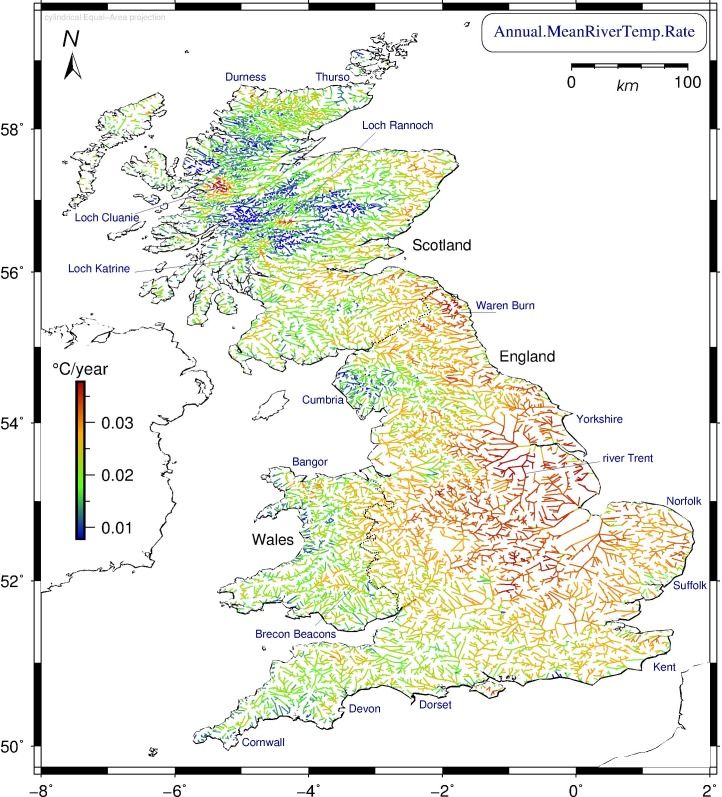
Modelled rates of change in annual mean water temperature for Britain’s rivers in 1982–2011. The 20,578 British river segments show large geographical differences in warming response. Central England, the east coast and Yorkshire regions experienced the highest rates, whereas Cumbria and parts of Wales have the lowest. The Scottish Highlands show lowest annual warming response overall, but with notable exceptions (lochs Cluanie, Rannoch, and Katrine) that reflect local anomalies in air temperature there. Cylindrical equal-area projection. Scottish model results extrapolate English and Welsh data.

The mean annual river water temperature across all British freshwater segments was 9.84°C (+/- 0.038°C), rising from 9.62°C in the first decade (1982–1991) to 10.07°C in the last (2002–2011). However, these values merely hint at the true complexity of Britain’s warming rivers. Fig 2 shows the annual mean warming rates per river segment, highlighting significant regional differences (mean 0.22°C/decade, or 0.02°C/year on average; the 95%-confidence interval ranges from +0.08 to +0.35°C/decade). Eastern parts of England experienced greatest warming, whereas elevated parts of Wales, Cumbria, and most of the Scottish Highlands displayed least, but with important exceptions. Contrasting Figs 1 and 2, we observe that the warmest rivers over the studied three decades do not typically coincide with the ones that are changing most rapidly, suggesting that future epochs will see important shifts in regional thermal regimes (see below).

**Fig 3:**
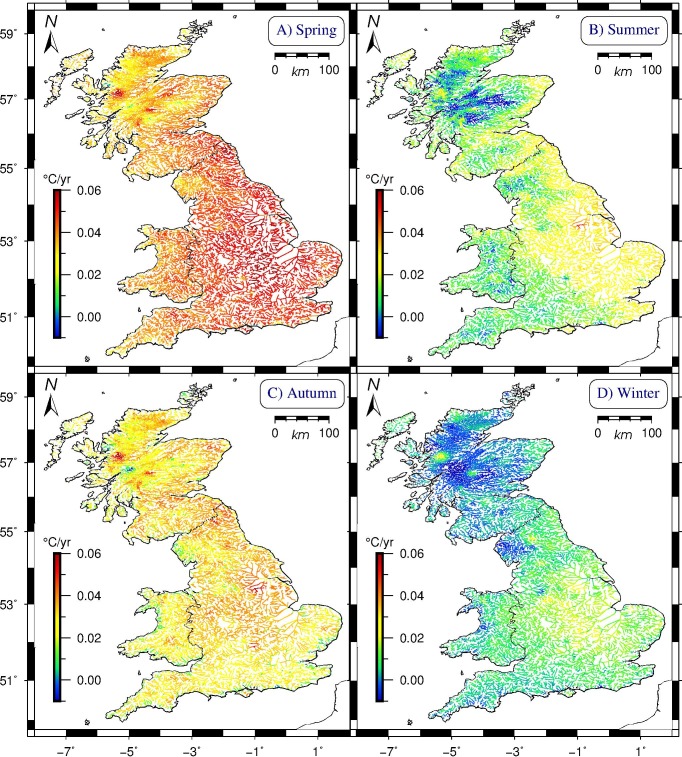
Modelled rates of change in seasonal mean water temperature for Britain’s rivers in 1982–2011. (A) the meteorological spring displays the highest mean warming rate (mean: +0.041°C/yr) in rivers throughout Britain; (B) Summer (mean: +0.013°C/yr) has the broadest range of rates, from -0.048 to +0.050°C/yr; (C) Autumn’s warming rates (mean: +0.031°C/yr) are lower than Spring’s, but higher than in Summer; (D) Winter is anomalous, with large parts of Britain exhibiting freshwater cooling, and a mean warming rate only marginally above zero. Cylindrical equal-area projection. Scottish model results extrapolate English and Welsh data.

Table 5 lists the mean annual warming rates over all segments for specific parts of the year. The accuracy and range of these estimates is expressed in their standard error (SEM, lower is better) and standard deviation (StDev) respectively. Comparing the four meteorological seasons, spring has the highest warming rates (mean: +0.41°C/decade, two-standard deviation range: +0.26 to +0.57°C/decade), followed by autumn (+0.30°C/decade, +0.16 to +0.45°C/decade), and, at a distance, by summer (0.13°C/decade, -0.07 to +0.32°C/decade) and winter (0.05°C/decade, -0.10 to +0.19°C/decade). Geographically, consistently high warming is found throughout the seasons around three Scottish lochs, the area southeast of Bangor in Wales, and the Waren Burn in Northumberland and the river Trent, both on England’s east coast (Fig 2). Most seasonal variability in the mean rates of warming, on the other hand, is observed in Cornwall, Norfolk, Suffolk, Kent, the Brecon Beacons in south Wales, and along the northern shores of the Scottish Highlands, from Durness to Thurso. Season-specific warming rates per segment are plotted in the four panels of Fig 3 (with fixed colour scale, highlighting differences between the seasons).

**Table 5:** Statistics of British river segment annual warming rates in °C/yr for 1982–2011.

Period	Mean Rate	SEM	StDev	Min	Max
Annual	0.0216	4.9e-5	7.026e-3	-0.0279	0.0516
Spring*	0.0414	5.5 e-5	7.882e-3	-0.0159	0.0707
Summer*	0.0125	6.9 e-5	9.871 e-3	-0.0480	0.0503
Autumn*	0.0305	5.2 e-5	7.451 e-3	-0.0258	0.0672
Winter*	0.0045	5.1 e-5	7.262 e-3	-0.0372	0.0307
Jan	0.0199	5.9 e-5	8.527 e-3	-0.0315	0.0481
Feb	0.0402	1.1e-4	1.574 e-2	-0.0172	0.0754
Mar	0.0242	4.5 e-5	6.413 e-3	-0.0197	0.0503
Apr	0.0627	6.2 e-5	8.869 e-3	0.0014	0.0958
May	0.0380	8.6 e-5	1.228 e-2	-0.0293	0.0729
Jun	0.0272	9.1 e-5	1.299 e-2	-0.0478	0.0689
Jul	0.0004	8.1 e-5	1.012 e-2	-0.0688	0.0376
Aug	0.0105	6.0 e-5	8.654 e-3	-0.0446	0.0448
Sep	0.0406	7.5 e-5	1.077 e-2	-0.0178	0.0853
Oct	0.0231	5.3 e-5	7.668 e-3	-0.0291	0.0580
Nov	0.0280	5.9 e-5	8.415 e-3	-0.0303	0.0678
Dec	-0.0412	4.9 e-5	6.975 e-3	-0.0771	-0.0046

Sample size: 20,578 segments per row. Listed mean rates per year are a factor of ten smaller than the decadal rates in the main text. SEM = standard error of the mean.

* = meteorological season

Assessing rates of change for individual months, April and December are most extreme, but with opposite sign. Over the studied thirty-year period, British rivers have on average warmed by almost two full degrees centigrade in April (+0.63°C/decade, two-standard deviation range: +0.45 to +0.80°C/decade). By contrast, December rates evince strong cooling throughout Britain (-0.41°C/decade, range: -0.55 to -0.27°C/decade). This cooling is most pronounced in the Scottish Highlands and the Lake District (Cumbria), and least along all coasts. In addition, mountainous areas (in the Scottish Highlands, Cumbria, and Wales) show cooling of circa -0.1 to -0.2°C/decade in July and August. Cooling is also observed in the southern counties of Cornwall, Devon, and Dorset in July. Elsewhere, these summer months register the weakest positive rates, yielding global monthly warming means of just above zero for July and +0.10°C/decade for August. The remaining months can be broadly divided into a faster-warming group (May, Sep, Feb; from +0.40 to +0.38°C/decade) and ones with modest mean warming rates (Nov, Jun, Mar, Oct, Jan; from +0.28 to +0.20°C/decade). Lastly, substantial warming is seen throughout the Scottish Highlands in April and September, and in Cornwall in October and November. Month-specific warming rates per segment are plotted in Figures H-S in S1 File.

**Fig 4:**
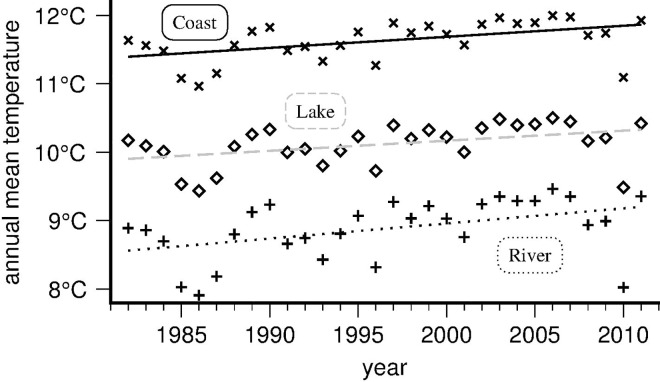
Annual mean warming of Britain’s rivers (1982–2011), per river segment class. Warming for riverine (plus symbol, mean rate: +0.221°C/decade), lacustrine (diamond, +0.147°C/decade) and coastal segments (cross, +0.161°C/decade); error bars are too small to plot. Coastal segments are warmest, but river segments are warming fastest.

Analysed per class, coastal segments show the highest absolute mean temperatures (11.63°C +/-0.053°C), followed by lakes (10.11°C +/-0.056°C) and inland rivers (8.88°C +/-0.080°C). But in terms of warming (Fig 4), the riverine mean rate (+0.22°C/decade) exceeds both the coastal and lacustrine rates (at +0.16 and +0.15°C/decade respectively). However, all remain well below the mean rate of increase for air temperature over Britain for the studied period (+0.31°C/decade).

Aside from hydrological [7, 11, 29–30] and economic [10] implications of these results, the impacts of derived and projected trends are of interest to aquatic ecosystems, water quality, aquaculture, wild fish, and fisheries. Cold-stenothermic species such as Atlantic salmon (*Salmo salar*), various subspecies of trout, and Arctic Char (*Salvelinus alpinus*) could experience a myriad of challenges. These may involve spawning, embryonic development, hatching, emergence, age at first maturity, fecundity, olfaction, behavioural and physiological changes, longevity, habitat shift and reduction, and indirect detrimental effects (lake eutrophication, reduced resource availability, stress). For anadromous species, the age at smolting and the timing of seaward migration and subsequent freshwater re-entry could also be affected [13, 27, 31, 32–39]. Even though salmon may have some capacity to physiologically adapt to higher water temperatures when given sufficient time to evolve, current upper tolerance limits for salmon and trout will (given the observed and modelled rates) likely be exceeded locally within the next few decades, which may cause significant shifts in salmonid habitat and phenology [40–44].

**Fig 5:**
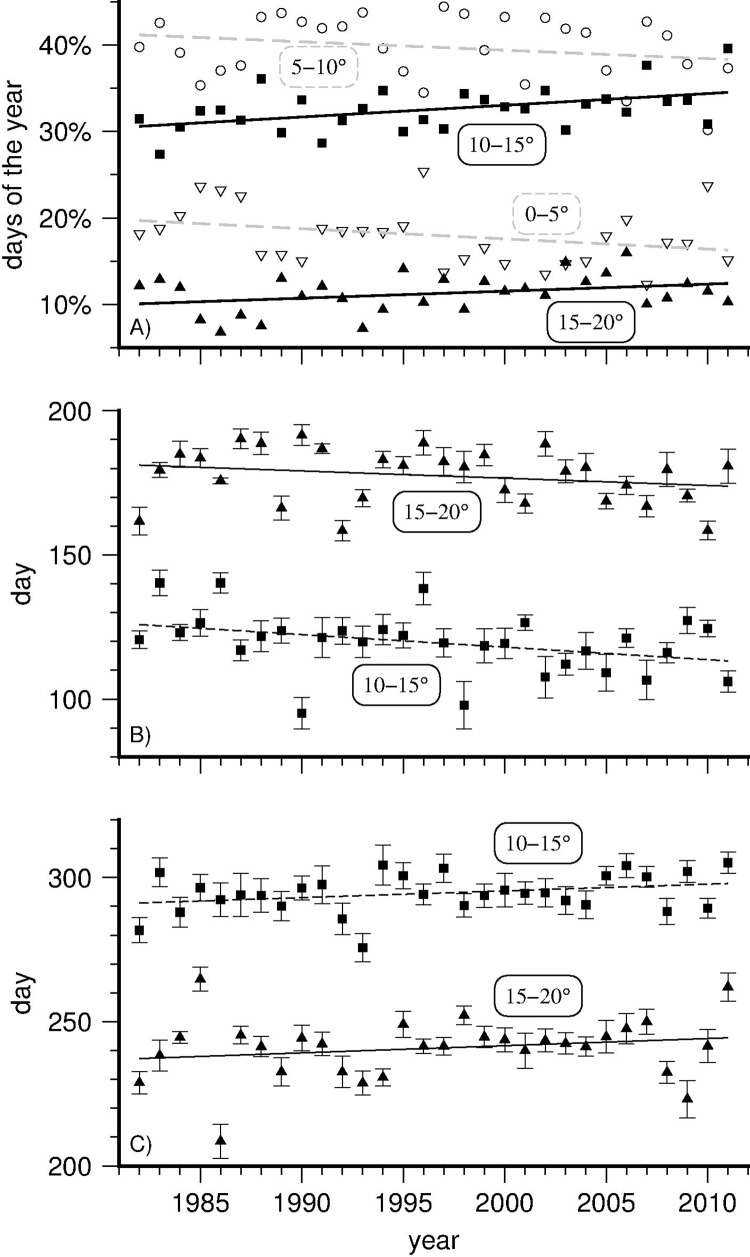
The time Britain’s rivers spent in five-degree temperature brackets (1982–2011). (A): proportion of the year during which river segments reside in each five-degree temperature bracket: 0–5°C (open downward triangle); 5–10°C (open circle); 10–15°C (solid square); 15–20°C (solid upward triangle); the two warmer brackets increase in time span(solid line fits), whereas the two colder ones decrease (dashed line fits); (B,C): mean day of the year (1–365) on which segments cross the lower bound of the two highest brackets, resp. first entering (in panel B) and finally leaving (in panel C); both dates shift outward at similar mean rates; error bars: +/- standard error of the mean.

To illustrate this, Fig 5A displays the changing widths of four five-degree temperature brackets, showing that British river segments spend on average ever more days in the topmost two brackets (spanning 10–20°C), with well over 30% of the year spent in the 10–15°C bracket alone. Moreover, Fig 5B and 5C evince that segments tend to enter the water temperature range above 10°C earlier in the year and leave it later in the year than in the past, again identifying spring and autumn as the seasons subject to most change. This translates into some specific challenges for fish. At the low end of the scale, water temperatures below 10°C are considered optimal for salmonids during the spawning season [27], and this window has on average narrowed by about two weeks in spring (and by another week in autumn). At the other end of the scale, freshwaters above 20°C may create thermal barriers that reduce the number of upstream swimming adult salmon returning from the sea [27].

Furthermore, in terms of parasitic and pathogenic fish disease outbreaks, the intermediate temperature bracket of 10–15°C is of particular concern. Invasive exotic pathogens aside, several endemic diseases of salmonids clinically express above a threshold temperature in this temperature range, likely becoming longer-lasting and more prevalent, severe, and widely spread as this bracket expands in duration. Other, more incidental viral threats also tend to survive longer, express more, or produce higher mortalities when water temperatures range between 10–15°C [45–51]. The geographical distribution of segments that spend most time in this temperature bracket in 1982–2011 is plotted in Figure T in S1 File.

## Discussion

The three main aims of this study were 1) to derive a numerical model of daily mean freshwater temperature in mainland Britain for the period 1982–2011 that incorporates the most important topographical and time-dependent factors; 2) to reconstruct daily mean river water temperatures for each individual British river segment, including geographical extrapolation to Scotland; and 3) to quantify warming trends per river segment so as to identify how and where river water temperatures have changed in the past and may do so in future. Here we briefly assess the resultant model’s main merits and limitations, compare the overall warming rates we found with those reported in recent literature, and touch upon future projected warming.

Our model has enabled the geographical extrapolation of a parametrisation of water temperature data in England and Wales to estimate water temperature in Scotland. Thus the model provides complete coverage for mainland Britain, giving detailed information for every river segment at any day over the period 1982–2011. Moreover, freshwater predictions are not restricted to the currently-employed ECRINS segment definitions; in theory, one could supply parameter ensembles for other locations too; for example, if a higher-resolution description of Britain’s river catchments were used, or if a new canal, fish farm, or recreational fishery is created. As long as the segment class, coordinates, elevation, slope, sea distance, and recent air and sea temperatures are available, the model can generate a quantified estimate. Another key advantage is that the existing empirical data suffer from extreme outliers and incomplete, variable coverage; the model results resolve both of these issues.

The daily mean water temperatures produced by the model per river segment have not been post-processed in any way. Since their dominant inputs are air temperatures, which can be highly variable on short timescales, they may fluctuate more than daily time series of observed freshwater temperatures such as those in the SWTA data set. This suggests that some form of smoothing could possibly reduce model residuals further. However, we have not applied any smoothing of these time series ourselves. The model output is, furthermore, well-behaved in the sense that it does not suffer from data outliers. However, the presented model is emphatically not intended to supplant, or detract in any way from the value of original measurements, but rather, to provide a single comprehensive reconstruction at the level of individual river segments wherever and whenever direct observations are unavailable.

Regarding the limitations of this effort, the presented model should be considered preliminary in some respects. The most important one is the lack of Scottish data coverage, which has likely degraded model performance there. Features specific to some parts of the Scottish Highlands (e.g., localised effects of snow cover, steep valley-channelled winds, ground frost, and narrow, elongated lochs) may not have been adequately captured, and larger error margins should be expected if and when extensive data sets of freshwater temperature measurements above 56° N become available for comparison. However, air and sea temperatures are by far the largest contributing factors to our model’s river temperature estimates (Table 3), and for these inputs we do have complete coverage at the same high resolution and accuracy throughout Britain.

Another caveat concerns the effects of large water bodies. Wind direction and strength may cause other SST grid cells than the nearest one in the Euclidean sense (as used here) to affect particular inland locations. The incorporation of daily recorded wind vectors may therefore improve future modelling efforts. Similarly, using the distance to the nearest lake centroid constitutes a conscious oversimplification of actual geography. This could be addressed in future work by instead evaluating the distance to the nearest lake shoreline, using ECRINS’s database of lake polygons.

It is furthermore worth observing that several other potentially relevant factors are absent in our model. These include rainfall, prevailing wind direction and strength, snow cover, the amount of riparian shade [5, 7, 52], bathymetry, discharge volume, flux dynamics, and human water management, including both industrial and agricultural influences. Some of these data might become publicly accessible for the whole of Britain at the required resolution in future. With respect to rainfall, however, [53] note that annual mean precipitation over England and Wales has not changed significantly since records began in 1766. Thus, annual precipitation may not be relevant for multi-decadal warming rate analyses), but seasonal rainfall, on the other hand, is highly variable [11].

Regarding discharge, debate is still ongoing regarding its relevance for freshwater temperature modelling. For example, [4] recommend its inclusion in future models, but [2] cite the SWTA observational record to argue that changes in river flow have had little influence on observed water temperature trends. [12] further note that low flows can greatly reduce thermal capacity, which could introduce bias in trend analyses [54]. Thus future studies may need to explore this issue in more detail, in order to quantify the possible significance of discharge effects.

A survey of recent literature on the subject of British freshwater warming rates provides both correspondences and contrasts. The freshwater warming rates we found are lower than those reported for English and Welsh river sites in the SWTA by [2] and [26], at +0.30 and +0.29°C/decade respectively. This difference is due to our inclusion of Scotland, where mean rates are on average lower. Regarding lake temperatures, recent work [28] reports global rates over 1985–2009 of +0.34°C/decade (and +0.12°C/decade for oceans, +0.25°C/decade for air globally), whereas [31] looked at data for twenty-four European lakes and found 0.15–0.30°C/decade. Contrastingly, [16] found far lower rates for U.S. rivers (up to +0.09°C/decade).

Regional extremes in British warming and cooling of river waters (such as Scottish warming in April and southwestern cooling in July) correlate highly with extremes in local air temperature. Freshwater extremes tend to be lower than those for air temperatures, likely due to maritime effects, topography, discharge dynamics, evaporation, and latent heat [12, 46]. By contrast, the SST around Britain displays less warming over the studied three decades than observed in air temperatures (see Figure A in S1 File). Another factor of interest concerns snow cover; [52] reported that rain-dominated, low-elevation catchments were found to be far more sensitive to air temperature variations than streams draining steeper topography whose flows were dominated by snowmelt. A warmer climate that causes a larger proportion of precipitation to fall as rain rather than snow in Scotland could thus increase the sensitivity of Scottish rivers to ambient temperatures in future.

**Fig 6:**
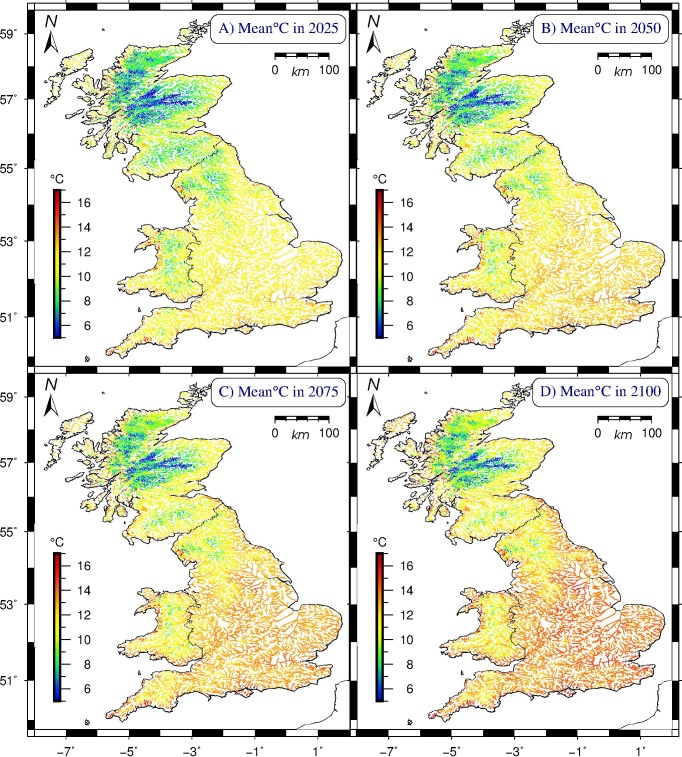
Modelled mean British water temperatures per river segment in four future epochs. All projections are based upon linear fits of modelled annual means per segment over 1982–2011. (A) Mean freshwater temperatures for the year 2025 (overall mean 10.36°C, two-standard deviation range: 5.29–15.65°C); (B) projections for 2050 (mean 10.87°C, range: 6.07–15.67 C); (C) projections for 2075 (mean 11.37°C, range: 6.45–16.28°C); projections for 2100 (mean 11.87°C, range: 6.82–16.93°C). Cylindrical equal-area projection. Scottish model results represent a geographical extrapolation of English and Welsh data.

The model also allows us to make temporal extrapolations beyond the three decades of water temperature measurements, by extending the linear fits of annual means per segment to future years (see Text B in S1 File). This enables us to make model-based predictions on how the river temperatures may change in the future. As future data are released, the accuracy of these predictions can be ascertained. Fig 6 displays four epochs in the 21^st^ century at 25-year intervals (with fixed colour scale). In terms of additional degrees centigrade of warming per segment with respect to their individual mean over 1982–2011, the total distribution over the 21^st^ century both flattens and moves rightward (Figure U in S1 File), shifting the overall mean by +2.23°C by the year 2100, with the +/-2 standard deviation range of additional mean freshwater temperatures (with respect to individual segment means over the studied period) then spanning +0.78 to +3.69°C.

Lastly, this approach may be repeated in the future for Britain or elsewhere. In contrast to the MetOffice’s UKCP09 project, which has now finished, satellite-based measurement of global SST is still ongoing; the latest, gridded results usually become available online within a day after observation. Thus if in the future, the MetOffice releases additional years of gridded daily mean air temperatures beyond 2011, current river water temperature predictions could be extended, using either the existing model or a more refined one. Further afield, the ECRINS database covers 48 European countries, enabling similar investigations to this one throughout Europe wherever time series of daily air temperatures and a sufficient sample of freshwater temperature measurements are available.

## Conclusion

The river water temperature model we constructed for mainland Britain on the basis of four large, empirical data sets has allowed us to quantify several distinct features, both geographically and over time. In particular, it enabled the generation of a complete database of estimated daily mean water temperatures for every British river segment, whether it was measured or not. It also enabled geographical extrapolation to Scotland, for which no published data were available to us. Temporal extrapolation beyond the database furthermore enabled projections into the future to determine the likely impact on Britain’s rivers throughout the 21^st^ century. We summarise the main findings below.

- Britain’s mean river water temperature over the studied period (1982–2011) was 9.84°C (Fig 1); the mean warming rate was 0.22°C/decade; England’s southern and eastern regions warmed most over the modelled interval, whereas elevated parts of Wales, Cumbria, and most of the Scottish Highlands warmed least (Fig 2).
- Warming rates for specific seasons and months (Fig 3, Table 5) show large differences, with spring and autumn subject to most warming, especially the month of April, warming by +0.63°C/decade on average over the studied period; by contrast, December rates evince strong cooling throughout Britain of -0.41°C/decade on average, most pronounced in the Scottish Highlands and the Lake District (Cumbria), and least along all coasts.
- British river segments spend on average ever more days at temperatures above 10°C, with well over 30% of the year in the 10–15°C bracket, considered most conducive to large outbreaks of fish diseases; segments tend to enter (leave) this bracket earlier (later) in the year, again with spring and autumn being the seasons subject to most change (Fig 5).
- Our model predicts that by the year 2100, Britain’s rivers will on average have warmed by +2.23°C with respect to their 1982–2011 mean temperature, with individual river segments on average adding between 0.78 to 3.69°C to their annual mean (Fig 6).

To facilitate access to the modelling results, we have stored all model-estimated daily mean water temperatures as individual time series (1982–2011) for each British river segment in a public online repository [21]. Ancillary metadata describe the ECRINS-defined segments in more detail. The four data sets in Table 1 used in creating the models are, moreover, accessible from the websites of the agencies that collect(ed) them (see the web links in the references) [22–25].

To effectively inform both the surveillance of wild and managed fish populations, water quality, targeted mitigation responses to outbreaks of waterborne pathogens, and society’s own reliance on freshwater resources, assessments of freshwater temperature need to consider the time-variant spatial heterogeneity of Britain’s warming rivers. Widespread, continuous collection of such data is therefore of paramount importance. Wherever and whenever such measurements are missing, however, the river water temperature model we derived may serve as an alternative estimate of local freshwater temperature, based upon its derived general relationships with the physico-geographical properties of British river segments, ambient air temperatures, and nearby sea surface temperatures. It constitutes not just a high-resolution quantified profile of British river segment characteristics over the past few decades, but also lays the foundation for projections of local future warming that may provide food for thought for hydrologists and environmentalists, as well as policy makers, the aquaculture industry, and other stakeholders.

## Supporting Information

S1 File Supplementary Materials. Data Description (Text A), Technical Discussion (Text B), and Supplementary Figures A-U.
(PDF)
