# Supplementary material for: The Differential Warming Response of Britain’s Rivers (1982–2011)
Source: PLoS One. 2016 Nov 10;11(11):e0166247. doi: 10.1371/journal.pone.0166247 (PMC5104490; doi:10.1371/journal.pone.0166247)
Supplement: S1 File — Data Description (Text A), Technical Discussion (Text B), and Supplementary Figures A-U. (PDF) [file pone.0166247.s001.pdf]

Supplement to:

The differential warming response of Britain's rivers (1982-2011)

ART Jonkers & KJ Sharkey (PLOS ONE, 2016).

Text A. Data Description

In June 2012, the European Environment Agency (EEA) released version 1.1 of the European Catchments and Rivers Network System (ECRINS), a geographical information system (GIS) of the European hydrographical environment with full topographical information, documented extensively in EEA Technical report 7/2012. Both the full data sets (aggregation catchments, rivers, lakes, functional elementary catchments (FECs), nomenclature, and ancillary data) and the report are freely available online from the ECRINS website [22]. These data resources were compiled using information from 1990-2006, and cover a total of 1,348,163 river segments in the following countries: Albania, Andorra, Austria, Belarus, Belgium, Bosnia and Herzegovina, Bulgaria, Croatia, Cyprus, the Czech Republic, Denmark, Estonia, Finland, France, Georgia, Germany, Greece, Hungary, Iceland, Ireland, Italy, Kazakhstan, Kosovo, Latvia, Liechtenstein, Lithuania, Luxembourg, Macedonia , Malta, Moldova, Monaco, Montenegro, the Netherlands, Norway, Poland, Portugal, Romania, Russia, San Marino, Serbia, Slovakia, Slovenia, Spain, Sweden, Switzerland, Turkey, Ukraine, and the United Kingdom. We primarily used the River data sets, with minor contributions from Lakes (for classifying nearby segments as lacustrine) and the Gazetteer (nomenclature).

In order to extract the subset of British mainland river segments, we loaded the source data into the QGIS software package (http://www.qgis.org/), identified the relevant data layer, and converted ECRINS's native Eurocentric coordinate system (EPSG:3035, i.e., a Lambert Azimuthal equal-area grid centred upon 52° N, 10° E, WGS84 ellipsoid) to a regular latitude/longitude grid (EPSG:4326, WGS84). We then selected the designated region of mainland Britain and calculated Northing and Easting in the United Kingdom's Ordnance Survey grid. Once extracted, we associated additional information with the 20,578 British river segments (comprising 22,088 unique nodes, and 1,482 catchments) from the nodes' descriptions (e.g., node elevation), the lake centroids, and the other main data resources used in this study.

The second dataset concerns air temperatures. The United Kingdom's meteorological office (or "MetOffice") compiled the UKCP09 gridded data sets to encourage and facilitate research into climate change impacts and adaptation. To quote from the MetOffice website: "The data sets cover the UK at 5 × 5 km resolution and span the period 1914–2011. They are available for daily, monthly, and annual timescales, as well as long-term averages for the 1961–1990 climate baseline period. Baseline averages are also available at 25 × 25 km resolution (to match the UKCP09 climate change projections) and as regional values." More details on format, accuracy, processing, and quality control can be found at the website [23]. Downloading the data (subject to non-commercial government license) requires free pre-registration and compliance with specified terms and conditions.

The U.S. National Oceanic & Atmospheric Administration's (NOAA) data set of daily optimum interpolation (OI, done by NOAA) of sea surface temperatures (SST, version 2, est.2009; grid resolution: one quarter-degree) is documented in [20, 24], and can be downloaded as grids. This is the third data set we used. For the spatial bounds of 49-62 N, 8 W to 3 E, NOAA provides daily grids of 44 x 52 cells (of which 1,637 at sea contained SST data), spanning latitudes 49.1 to 61.9 N, 7.9 W to 2.9 E, which covers our area of interest. For each sea cell we computed its centroid's northing and easting to identify the nearest SST grid cell for each British river segment in the ECRINS database. Fig A plots annual mean warming rates per grid cell for observed British air temperatures (AT, mean 0.031 ^{o}C/year, with large regional differences) and SST (4.45e-4 ^{o}C/year). This shows that sea surface temperature warming around Britain was negligibly small over the studied interval. (All supplementary figures can be found at the end of this document.)

The last data set we used concerns historical measurements of British river and lake water temperatures, collected since the 1950s in England and Wales. In 2012, the United Kingdom's Environment Agency, in collaboration with the Countryside Council for Wales, published the "Surface Water Temperature Archive" (SWTA), which has dense spatial coverage from the early 1980s onward [2, 26-27]. These data can be downloaded directly from http://www.geostore.com; they comprise over 29 million observations spanning the period 1954-2007, the majority of which covers the last three decades.

The full SWTA archive contains data for rivers (28,031 sites), canals (789 sites), coastal waters (268 sites), lakes (966 sites), estuaries (457 sites), and drains (57 sites). Reference [25] interprets climate change impacts and provides background, description, and interpretation of this data set, as do [2, 19, 26-27]. The SWTA

classifies observation sites as of type river, lake, or coastal, which are all-inclusive and mutually-exclusive (that is, each site is classified as being exactly one of these three types). In addition, two other categories, transitional and groundwater, are sometimes added to the SWTA metadata, but this applies only to a minority of sites. For example, in the studied interval, only 11 sites (all in East-Anglia) have a "groundwater" classification, in addition to being of "river" type. It would have been interesting to explore the groundwater class separately, but their representation is temporally and spatially insufficient to produce reliable models. In terms of the time of observation, the large majority of measurements was collected by data loggers, providing equal sampling (flat frequency distribution) for different times of the day and night. The remaining spot measurements account for only 9.71% of the total, and peak between 11 a.m. and noon, several hours before the hottest time of day (3-4 p.m.) The fewest measurements were taken between 5-6 a.m, the coldest time of day. Thus both minimum and maximum temperature are relatively poor representatives of the full sample, supporting our choice of the mean as a better representation of daily temperature.

Text B. Technical Discussion

Modelling Methodology

Our modelling approach first fit for each class separately (coastal, lacustrine, riverine), an initial set of all frequency-weighted daily means of SWTA measurements spanning the central two-decades (1987-2006) of our three-decade target period of 1982-2011. These three decades are bounded by the starting year of the SST dataset and the final year of the UKCP09 dataset. Thus each model consists of three separate sub models, one for each segment class. As the number of riverine-classed observations exceeded those of the other classes by two orders of magnitude, we investigated sub-sampling the riverine set at 1%, and compared models based upon this subset and using the full set, finding no statistically significant differences.

We then explored various partial and iterative weighted least-squares modelling techniques using both standard statistical software (Minitab v.16) and custom-written code using the Eigen linear algebra template library (http://eigen.tuxfamily.org/). The main challenge here was that the SWTA dataset consists of raw data without extensive quality controls, causing extreme outliers (for example, due to typographical error) which significantly degrade initial model fits, even when using daily means per site. We therefore explored iterative clamping of the most extreme outliers (that is, removing individual mean temperatures for a particular day at a particular site) in conjunction with successive re-modelling passes. This is a technique borrowed from geophysics.

With respect to the original full sample size, all outlier rejection passes together typically removed 5-7% of individual daily means as outliers; in the most extreme case, 8.9% of the initial data were classified as outliers, given a multi-step +/-2σ bound (sigma = standard deviation). As an example, Table A lists the parameter coefficients, adjusted R^2 (sometimes called the "coefficient of determination") and sample size reduction due to outlier rejection for the lacustrine segment class. In this case, 4.82% of the raw data are rejected when a single two-sigma cut-off is imposed, whereas multi-step rejection removes 8.58% over all steps combined. Most coefficients differ only marginally between the two schemes, but the amount of data variance captured by the model increases an additional 1.6% when multi-step outlier rejection is applied.

The final model enabled us to generate time series of daily mean freshwater temperature at any location within mainland Britain for which the requisite model inputs are available for the desired segment class. For the British river segments defined in the ECRINS dataset, these are freely available for download [21]. Comparative statistics with regard to the SWTA data set are shown in Figs D-E; boxplots of seasonality-related descriptive statistics can be found in supplementary Figs F-G.

The generated time series per segment also allowed us to compute the mean temperature per year over the studied interval, yielding thirty data points for each of the 20,578 river segments. Annual warming rates were then obtained by computing the slope of a least-squares fitted line through these annual points per segment. The line coefficients (intercept and slope) in turn provided segment-specific predictors of the mean annual water temperature for any desired future year. Mean future water temperature for the 21st century at 25-year intervals are plotted per segment in Fig 6 in the main text; histograms of their distribution are plotted in Fig U.

Table A. Derived model coefficients for the lacustrine segment class, based upon raw data, and after single-pass c.q. multi-pass outlier removal at +/-2 standard deviations.

Parameters	Raw Means	Single-pass	Multi-pass
Constant	0.61471	0.37761	0.30408
Elevation	5.0557e-3	2.9673e-3	1.9857e-3
SeaDistance	-1.9381e-2	-1.4428e-2	-1.2576e-2
SeaTemp0	1.9636e-1	2.2586e-1	2.6374e-1
SeaTemp1	3.1629e-1	3.1230e-1	2.8025e-1
AirTemp0	9.9563e-2	1.0022e-1	1.0314e-1
AirTemp1	6.6658e-2	06.7003e-2	6.5541e-2
AirTemp2	6.4820e-2	6.6929e-2	6.5078e-2
AirTemp3	5.4295e-2	4.5373e-2	4.9051e-2
AirTemp4	4.5031e-2	4.9938e-2	5.1148e-2
AirTemp5	4.4802e-2	4.5576e-2	4.6577e-2
AirTemp6	4.0801e-2	4.3417e-2	4.6846e-2
AirTemp7	3.5561e-2	2.9987e-2	2.6406e-2
AirTemp8	8.7782e-2	9.8206e-2	1.0143e-1
Adj. R-squared	85.8%	92.3%	93.9%
Sample Size	40,624	38,666	37,138
Sample redux	100%	95.18%	91.42%

Tuning a model to observations: goodness-of-fit, significance, and overfitting

In the canonical least-squares linear fitting example familiar from elementary statistics text books, a straight line is to be derived that best represents an elongated point cloud in a two-dimensional Euclidean plane. Each point is defined by a coordinate pair of an independent X parameter, and its associated, dependent Y response variable. The line to be fitted is defined by a constant (the height at which it intersects the Y-axis, or “intercept”) and the X parameter coefficient, which represents the slope, that is, how response variable Y changes on average with increasing X. Provided that the number of points is greater than the number of coefficients (i.e., the system is overdetermined) and the problem is not ill-conditioned (e.g., all points do not coincide), a least-squares fit will derive those line coefficients (the model) that minimise the summed squared distances between each point and the line. The problem is easily extended to accommodate additional dimensions (more independent predictor variables), different weights associated with specific points (because some observations may be more reliable than others), non-Gaussian error distributions, binary, ordinal, or nominal variables, covariant inputs, and a non-linear response.

In the current case, a linear model is used to relate historical daily means of observed freshwater temperatures of variable quality and quantity (the response) to physico-geographical properties of a river segment and current and past ambient air and nearby sea surface temperature (the model parameters). Given a plethora of quantified segment properties to choose from, and long time series of past air and sea temperatures (inviting the question how far back in time the cut-off is to be placed), many different ensembles of parameters may produce model estimates that can all be deemed a “satisfactory” approximation of the available measurements in some sense. This leads us to the problem which one to choose. In the following examples, we use the full coastal class of segments (with 15,373 observed SWTA daily means over the three decades) to show how the members of a parameter ensemble were selected, and why these differ for different classes. All fits in this example are performed using the raw data, without any rejection of outliers. For illustration purposes, we start by creating a dummy variable that contains 15,373 random values drawn from a standard Gaussian distribution with zero mean and a standard deviation of unity. When fitting the real, non-random freshwater measurements to these random synthetics, the resulting least-squares model is obviously a complete failure. Nevertheless, we still gain some useful information from the statistics output (here printed in Courier font); values of particular interest are highlighted in red.

```
The regression equation is
TEMP_AVG = 12.9 - 0.0283 random

Predictor       Coef   SE Coef       T      P
Constant     12.8941    0.0360  358.26  0.000
random      -0.02829   0.03569   -0.79  0.428

S = 4.55881   R-Sq = 0.0%   R-Sq(adj) = 0.0%
```

The model’s goodness-of-fit (that is, how much data variance is accounted for by the model) is expressed in the (unadjusted and adjusted) R^2 statistic (R-sq = 0.0%, highlighted in red), whereas the mean observed temperature over all data is here recovered in the *Constant* (12.89 ^{0}C), with its standard error (SE Coef; smaller is better) quantifying its standard deviation, that is, how accurately the data estimate the coefficient’s unknown value. Dividing the coefficient by its standard error yields the *T* value. In combination with the number of degrees of freedom and the (known) *T*-distribution, this test statistic in turn yields the coefficient’s *p*-value (range: 0-1; smaller is better). For each coefficient, a null hypothesis states that the true coefficient is zero (has no effect), while the alternative hypothesis poses that a real effect is present, i.e., the true coefficient is non-zero. The *p*-value then expresses the probability of incorrectly rejecting the null hypothesis when it is actually true (the so-called type-I error), due to various sources of error, observational noise, or other issues. This can be seen in the example, in which the fitted coefficient for the random variable (the effect of which is known to be zero) is -0.028 (i.e., non-zero), but the associated *p*-value is 0.428, that is, there is a 42.8% likelihood that the derived effect is due to chance alone. This is generally considered an unacceptably high probability. In practice, a maximum acceptable *p*-value (the alpha level) is commonly set at 0.05 or lower, to limit the likelihood of this type of scenario. In the following outputs, reported *p*-values of “0.000” are smaller than 5.0e-4.

If a parameter that has no effect on the response variable is added to an existing ensemble of significant contributing parameters, the former’s own *p*-value immediately identifies it as such, as can be seen in the next example, in which some locally observed mean air and sea temperatures on previous days are added as model parameters:

```
The regression equation is
TEMP_AVG = 0.678 + 0.221 ST0 + 0.236 ST1 + 0.187 AT0 + 0.0827 AT1 + 0.0633 AT2
           + 0.0642 AT3 + 0.0369 AT4 + 0.121 AT5 + 0.0081 random

Predictor       Coef   SE Coef      T      P
Constant     0.67768   0.03639  18.62  0.000
ST0          0.22122   0.03048   7.26  0.000
ST1          0.23620   0.03002   7.87  0.000
AT0         0.187411  0.007313  25.63  0.000
AT1         0.082722  0.009372   8.83  0.000
AT2         0.063319  0.009479   6.68  0.000
AT3         0.064193  0.009557   6.72  0.000
AT4         0.036863  0.009434   3.91  0.000
AT5         0.120826  0.007417  16.29  0.000
random       0.00810   0.01112   0.73  0.466

S = 1.42012   R-Sq = 90.3%   R-Sq(adj) = 90.3%
```

Note that the R^2 is now high, representing the fact that the added real parameters are explaining some of the variation in the data. The random coefficient is now closer to zero, and the Constant no longer contains the mean temperature, because most of that value is now accounted for in the various daily temperature parameters. The *p*-values of all ensemble parameters except "random" are effectively zero; that is, these all provide statistically significant information for predicting the response variable. Compare this to what happens if we remove the random parameter from the model and extend the sequence of daily air temperatures another day further back in time:

```
The regression equation is
TEMP_AVG = 0.688 + 0.202 ST0 + 0.232 ST1 + 0.183 AT0 + 0.0854 AT1 + 0.0568 AT2
           + 0.0609 AT3 + 0.0442 AT4 + 0.0219 AT5 + 0.127 AT6

Predictor       Coef   SE Coef      T      P
Constant     0.68788   0.03603  19.09  0.000
ST0          0.20211   0.03020   6.69  0.000
ST1          0.23196   0.02972   7.80  0.000
AT0         0.182569  0.007245  25.20  0.000
AT1         0.085449  0.009281   9.21  0.000
AT2         0.056778  0.009393   6.04  0.000
AT3         0.060912  0.009466   6.44  0.000
AT4         0.044163  0.009351   4.72  0.000
AT5         0.021891  0.009269   2.36  0.018
AT6         0.126655  0.007246  17.48  0.000

S = 1.40623   R-Sq = 90.5%   R-Sq(adj) = 90.5%
```

Although the R^2 is even higher than before, one air temperature's *p*-value is now significantly larger than zero. Note that this p-value is not the one associated with the newly-added parameter AT6, but AT5. A similar effect occurs when extending the SST time series further back, for example, up to previous day 5:

```
 The regression equation is
TEMP_AVG = 0.649 + 0.216 ST0 + 0.0359 ST1 + 0.0716 ST2 + 0.0414 ST3 - 0.0428 ST4
           + 0.138 ST5 + 0.189 AT0 + 0.0830 AT1 + 0.0635 AT2 + 0.0637 AT3
           + 0.0367 AT4 + 0.119 AT5
```

Predictor	Coef	SE Coef	T	P
Constant	0.64911	0.03638	17.84	0.000
ST0	0.21596	0.03218	6.71	0.000
ST1	0.03591	0.05010	0.72	**0.474**
ST2	0.07165	0.05206	1.38	**0.169**
ST3	0.04143	0.05150	0.80	**0.421**
ST4	-0.04283	0.04904	-0.87	**0.383**
ST5	0.13829	0.03041	4.55	0.000
AT0	0.189391	0.007294	25.96	0.000
AT1	0.082966	0.009342	8.88	0.000
AT2	0.063536	0.009446	6.73	0.000
AT3	0.063735	0.009529	6.69	0.000
AT4	0.036744	0.009407	3.91	0.000
AT5	0.118863	0.007398	16.07	0.000

S = 1.41521   R-Sq = 90.4%   R-Sq(adj) = **90.4%**

As in the earlier case, R^2 is again excellent, but at the cost of significantly overfitting the model. Temperatures on consecutive days are likely to be covariant to some extent; therefore, some superfluous information weakens the response's dependency on similar parameters from the same sequence (note that the *p*-values of the AT parameters are unaffected in this latest example). Crucially, due to the nature of the matrix inversion underlying the model fitting process, this uncertainty can become mapped to *any* member of that subset, yielding potentially spurious information as to which parameters to excise. In this case, we know from earlier tests that parameter ST1 *does* carry valuable information with which to estimate the response, but this is now obscured by the redundant, extended ensemble.

Optimising a model thus requires a triple balance, between 1) the parameter and coefficient values themselves, 2) their individual *p*-values, and 3) the overall goodness-of-fit (R^2). Regarding the first aspect, the ECRINS database of segment characteristics contains many variables that have no bearing whatsoever on observed temperatures there, for example, a segment's azimuth (its compass orientation in the plane). A quick inspection of any of the maps in this paper will show that similarly-oriented river segments can be found in completely different regions and topographies, from the Scottish Highlands through the Midlands or Greater London to the Cornish coasts. The mere fact that a stream happens to flow in, say, a south-westerly direction does not inform the water temperature. In the fitting process, such parameters either produce a statistically significant coefficient of (close to) zero, or consistently yield unacceptably high *p*-values in all ensembles. Thus, identifying such null parameters is relatively easy. Likewise, if the parameter *values* themselves are by definition always (very close to) zero in a particular class (e.g., river slope of lake segments, sea distance of coastal segments), these can be excluded a priori from the exploratory model fitting process as well.

Recognising and preventing overfitting, however, requires more effort. A single fit can never convey sufficient information in this case, and a priori criteria may not be available to identify the optimum ensemble. Instead, every possible permutation of parameter combinations can be fitted separately, and their joint effects on *p*-values and R^2 tracked. In Table B, the time window of observed mean air and sea surface temperatures is progressively extended further back in time along each axis for up to one week; table cells contain R^2 and the number of non-zero *p*-values for each fit. The optimum (maximum R^2 *and* all *p*-values effectively zero) is found at six days of air temperatures and two days of SST (with day 0 = day of measurement to fit). This explains why different time windows were obtained for the other two river segment classes, where the relationships between observations, local topography, and time-dependent AT and SST are different. R^2 may be subsequently improved by the addition of (fixed-value) ECRINS variables that have non-zero coefficients *and* zero *p*-values, and by removing data outliers.

Table B. Goodness-of-fit (R^2) and number of overfitted parameters (*p*-value>5.0e-4) while extending air (rows) and sea (cols) temperature time series (AT#, ST#) back in time; the optimum (max. R^2 with no overfitting) is located at cell [AT0-AT5, ST0-ST1]. Coastal segments model, fitting 15,373 SWTA daily means.

	**ST0**	**ST0-ST1**	**ST0-ST2**	**ST0-ST3**	**ST0-ST4**	**ST0-ST5**	**ST0-ST6**
**AT0**	88.5/0	88.6/0	88.6/1	88.6/2	88.7/3	88.7/5	88.7/6
**AT0-AT1**	89.1/0	89.2/0	89.2/1	89.2/2	89.3/3	89.3/4	89.3/6
**AT0-AT2**	89.6/0	89.6/0	89.6/1	89.7/2	89.7/3	89.7/4	89.7/6
**AT0-AT3**	89.9/0	89.9/0	90.1/1	90.0/2	90.3/3	90.0/4	90.0/6
**AT0-AT4**	90.1/0	90.1/0	90.2/1	90.2/2	90.2/3	90.2/4	90.2/6
**AT0-AT5**	90.3/0	**90.3/0**	90.3/1	90.3/2	90.5/4	90.4/4	90.4/6
**AT0-AT6**	90.4/1	90.5/1	90.5/2	90.5/3	90.6/4	90.5/5	90.5/7

A related aspect concerns short-term autocorrelation in the parameter time series. A separate test model reduced the consecutive mean air temperatures to a single mean value over each class-specific time window of current and previous days of measurements (cf. [46]), producing highly similar, but marginally worse results across the board, yielding adjusted R^2 values ~2% below those for our own final model. The test model also had a higher outlier rejection rate (8.67% vs. 5.75%), with *p*-values for other parameters also being negatively affected (i.e., slightly higher) in this case, reducing confidence in the overall fit. We therefore concluded that using individual daily AT values was preferable to taking the means thereof. Nevertheless, we do value this test because, by definition, any autocorrelation present in time series of daily air and sea temperatures is here a priori removed by replacement with their mean. Thus its broadly similar performance to that of our final model shows that potential autocorrelation effects do not significantly degrade our chosen approach.

Finally, Table C illuminates annual and seasonal differences in the extent to which fluctuations in observed air and sea surface temperature correspond with one another and with those that the model estimates for inland freshwater temperatures over the period 1982-2011. Pearson correlations of the respective time series largely reflect the chosen optimum ensembles per segment class, in that correlations are highest for all seasons (0.89 to 0.96) between air and river temperatures. The model's correspondence with SST time series is more diverse, highest in autumn (0.80), closely followed by spring (0.74), whereas correlation is only moderate (0.51) in summer and negligible in winter (0.23). A similar pattern is evident in the seasonal correlations between observed AT versus SST. Thus the model can be seen to be affected more by AT than SST, as would be expected from the parameter ensembles that define the model (see Tables 2-3 in main text).

Given that sea surface temperature warming around Britain was negligibly small over the studied interval (see again Fig A), we expected freshwater warming to be less pronounced close to the coasts than far inland. Comparing the UKCP09 and SST data sets in terms of temporal variability for specific seasons, the two time series tend on average to be in closer agreement in spring and autumn than in summer and winter, during which SST tends to be less extreme than air temperature. This assessment is based upon the Pearson correlation of the two time series per meteorological season for each associated river segment (Table C), in which autumn's mean correlation of AT and SST is highest (+0.68), followed by spring (+0.59). By contrast, summer yielded on average only +0.31, and in winter, AT and SST appear not to correlate at all (mean correlation: +0.07).

Table C. Descriptive statistics of Pearson correlations per segment time series (1982-2011; sample size: 20,578 time series) between modelled river water temperature (this paper), observed local air temperature (MetOffice grid), and observed nearest Sea Surface Temperature (NOAA grid).

Time Series	Period	Mean	StDev	Min	Median	Max
River Water temperature vs. Local Air Temperature	Annual	0.960	0.022	0.846	0.967	0.975
	Spring	0.924	0.038	0.741	0.937	0.953
	Summer	0.887	0.064	0.607	0.910	0.930
	Autumn	0.940	0.028	0.797	0.950	0.961
	Winter	0.889	0.071	0.574	0.914	0.925
River Water temperature vs. nearby Sea Surface Temperature	Annual	0.859	0.045	0.731	0.858	0.972
	Spring	0.736	0.070	0.520	0.743	0.930
	**Summer**	**0.513**	0.128	0.249	0.516	0.897
	Autumn	0.797	0.094	0.608	0.799	0.945
	**Winter**	**0.225**	0.051	0.020	0.189	0.710
Local Air temperature vs. nearby Sea Surface Temperature	Annual	0.758	0.044	0.619	0.764	0.849
	Spring	0.588	0.058	0.395	0.605	0.709
	**Summer**	**0.310**	0.080	0.110	0.317	0.519
	Autumn	0.676	0.045	0.485	0.684	0.770
	**Winter**	**0.073**	0.033	-0.069	0.073	0.223

Example Derivation of freshwater temperature

Suppose we are interested in the freshwater temperature on the 31st of December 1986, for British segment object ID 84,225 (Catchment chain ID: 9,910), which is of class "Coastal" and has a 0.2 slope value (ECRINS field "pente"), with midpoint at latitude 50.03667 north and longitude 5.24724 west, or 167,501 Easting and 20,295 Northing in Ordnance Survey grid coordinates, which translates (through 1+int(<OS coordinate>/5000)) into MetOffice cell row 5, column 34 (the actual model parameters). This segment has a mean elevation of 13 meters above mean sea level, and is situated 12.681 km from the sea. Given the coordinates, we can also determine the nearest grid cell in the NOAA dataset of SST, for which we retrieve the sea surface temperature on day 0 (31 Dec 1986: 9.91 ^{o}C) and day 1 (that is, the *previous* day, 30 Dec 1986: 9.8 ^{o}C). In Table 3 in the main text, column Coast, we find (besides Constant = 0.55169) that northing, easting, pente, seaDistance, and AirTemp6-9 all have zero coefficients, so we can disregard these parameters for coastal sites. However, we do need air temperatures for days 0 to 5 (31st to 26th of Dec 1986, in that order, so counting backward in time from the chosen day of estimation). From the MetOffice's gridded data for the cell at row 5, column 34, we extract the daily mean air temperatures as 10.3 (on 31 Dec), 10.6, 10.1, 8.7, 7.5, and 7.8 ^{o}C, respectively. Now we can derive the model estimate for the freshwater temperature at the designated segment on the desired day, by taking the listed Constant for the coastal segment class, and adding to it the sum of the relevant parameters multiplied by their associated coefficients, as follows.

Freshwater temperature for segment 84225 on 31 Dec 1986 =

Constant + (Elevation * 0.028988) + (SeaTemp on day0 * 0.20697) + (SeaTemp on day1 * 0.27253) + (AirTemp on day0 * 0.16347) + (AirTemp on day1 * 0.076029) + (AirTemp on day2 * 0.062363) + (AirTemp on day3 * 0.062431) + (AirTemp on day4 * 0.042267) + (AirTemp on day5 * 0.137357) =

0.5517 + (13 * 0.028988) + (9.91 * 0.20697) + (9.8 * 0.27253) + (10.3 * 0.16347) + (10.6 * 0.076029) + (10.1 * 0.062363) + (8.7 * 0.062431) + (7.5 * 0.042267) + (7.8 * 0.137357) = 10.7 ^{o}C.

Supplementary Figure Captions

Figure A. Annual warming rates per grid cell over the studied interval (1982-2011), for Air Temperature (AT, per 5x5 km; mean 0.031 ^{o}C/yr) and Sea Surface Temperature (SST, per ¼-degree; mean: -4.45e-4 ^{o}C/yr).

Figure B. Out-of-sample hindcasts of the candidate model (1987-2006; grey lines) and SWTA observations (red dots) in the period 1982-1986, for several randomly-picked locations with over twenty-five years of data. Temporal resolution: 5 days.

Figure C. In-sample predictions of the final model (1982-2011; grey lines, five-day step) and SWTA observations (red dots) for the same locations as in the previous figure. Temporal resolution: 5 days.

Figure D. Final model's residuals for SWTA observational daily means per segment class and overall, after outlier removal. (A) Model residuals for coastal segment-associated SWTA daily means (13,627 points, mean: 0.0003 ^{o}C, standard deviation (StDev): 0.93 ^{o}C); (B) Model residuals for lacustrine segment-associated SWTA daily means (37,138 points, mean: 0.065 ^{o}C, StDev: 1.97 ^{o}C); (C) Model residuals for riverine segment-associated SWTA daily means (2,763,342 points, mean: -0.018 ^{o}C, StDev: 1.58 ^{o}C); (D) All residuals combined (2,814,107 points, mean: -0.017 ^{o}C, StDev: 1.59 ^{o}C). See also Table 4 in main text.

Figure E. Histograms of modelled (green) and observed (brown) annual warming rates for segments with 20+ years of SWTA measurements (daily means of raw data). *Left*: all 3,825 annual rates, bin width: 0.02 ^{o}C/yr. *Right*: subset range: -0.1 to +0.1/yr (3,459 annual rates), bin width: 0.005 ^{o}C/yr. The model greatly reduces the implausibly broad range of rates as derived from the (daily means of the) raw SWTA observations; a paired *t*-test for the means of the two data sets shows no significant differences (p=0.328), suggesting the model adequately captures the central tendency of observed annual warming.

Figure F. Boxplots of monthly mean (left panel) and standard deviation (right panel) over all 20,578 British river segments (1982-2011). Box marks interquartile range with midbar = median; whiskers (vertical line) mark upper (Q3 + 1.5 (Q3 – Q1)) and lower limit (Q1– 1.5 (Q3 – Q1)); stars = outliers. *Left*: July and August are the warmest months. *Right*: April (the fastest-warming month) displays least variability across Britain, whereas the winter months have most.

Figure G. Boxplots of annual, seasonal, and monthly warming rates over all 20,578 British river segments (1982-2011). Box marks interquartile range with midbar = median; whiskers (vertical line) mark upper (Q3 + 1.5 (Q3 – Q1)) and lower limit (Q1– 1.5 (Q3 – Q1)); stars = outliers. *Left*: Spring and Autumn are warming fastest. *Right*: April is the fastest-warming month; December is the only cooling month.

Figure H. Modelled annual warming rates (^{o}C /year) for British river segments in the month of January (1982-2011). Scottish model results extrapolate English and Welsh data.

Figure I. Modelled annual warming rates (^{o}C /year) for British river segments in the month of February (1982-2011). Scottish model results extrapolate English and Welsh data.

Figure J. Modelled annual warming rates (^{o}C /year) for British river segments in the month of March (1982-2011). Scottish model results extrapolate English and Welsh data.

Figure K. Modelled annual warming rates (^{o}C /year) for British river segments in the month of April (1982-2011). Scottish model results extrapolate English and Welsh data.

Figure L. Modelled annual warming rates (^{o}C /year) for British river segments in the month of May (1982-2011). Scottish model results extrapolate English and Welsh data.

Figure M. Modelled annual warming rates (^{o}C /year) for British river segments in the month of June (1982-2011). Scottish model results extrapolate English and Welsh data.

Figure N. Modelled annual warming rates (^oC /year) for British river segments in the month of July (1982-2011). Scottish model results extrapolate English and Welsh data.

Figure O. Modelled annual warming rates (^oC /year) for British river segments in the month of August (1982-2011). Scottish model results extrapolate English and Welsh data.

Figure P. Modelled annual warming rates (^oC /year) for British river segments in the month of September (1982-2011). Scottish model results extrapolate English and Welsh data.

Figure Q. Modelled annual warming rates (^oC /year) for British river segments in the month of October (1982-2011). Scottish model results extrapolate English and Welsh data.

Figure R. Modelled annual warming rates (^oC /year) for British river segments in the month of November (1982-2011). Scottish model results extrapolate English and Welsh data.

Figure S. Modelled annual warming rates (^oC /year) for British river segments in the month of December (1982-2011). Scottish model results extrapolate English and Welsh data.

Figure T. Modelled mean number of days per year river segments spent in the temperature bracket from 10 to 15 ^oC (the most high-risk for large outbreaks of fish diseases) over the studied interval of 1982-2011. The Scottish Highlands and southeast England were at lowest risk. Scottish model results extrapolate English and Welsh data.

Figure U. Histograms of modelled additional warming in ^oC per river segment for epochs 2025, 2050, 2075, and 2100 (A-D), relative to mean water temperature per segment over the period 1982-2011. Mean warming increases from +0.62 ^oC in 2025 to +2.23 ^oC in 2100, with an approximate Gaussian distribution around it (blue bell curve), which by 2100 ranges from below zero to over +4.5 ^oC of warming for individual segments; the +/-2 sigma range then spans +0.81 to +3.66 ^oC.

Supplementary Figures (A-U)

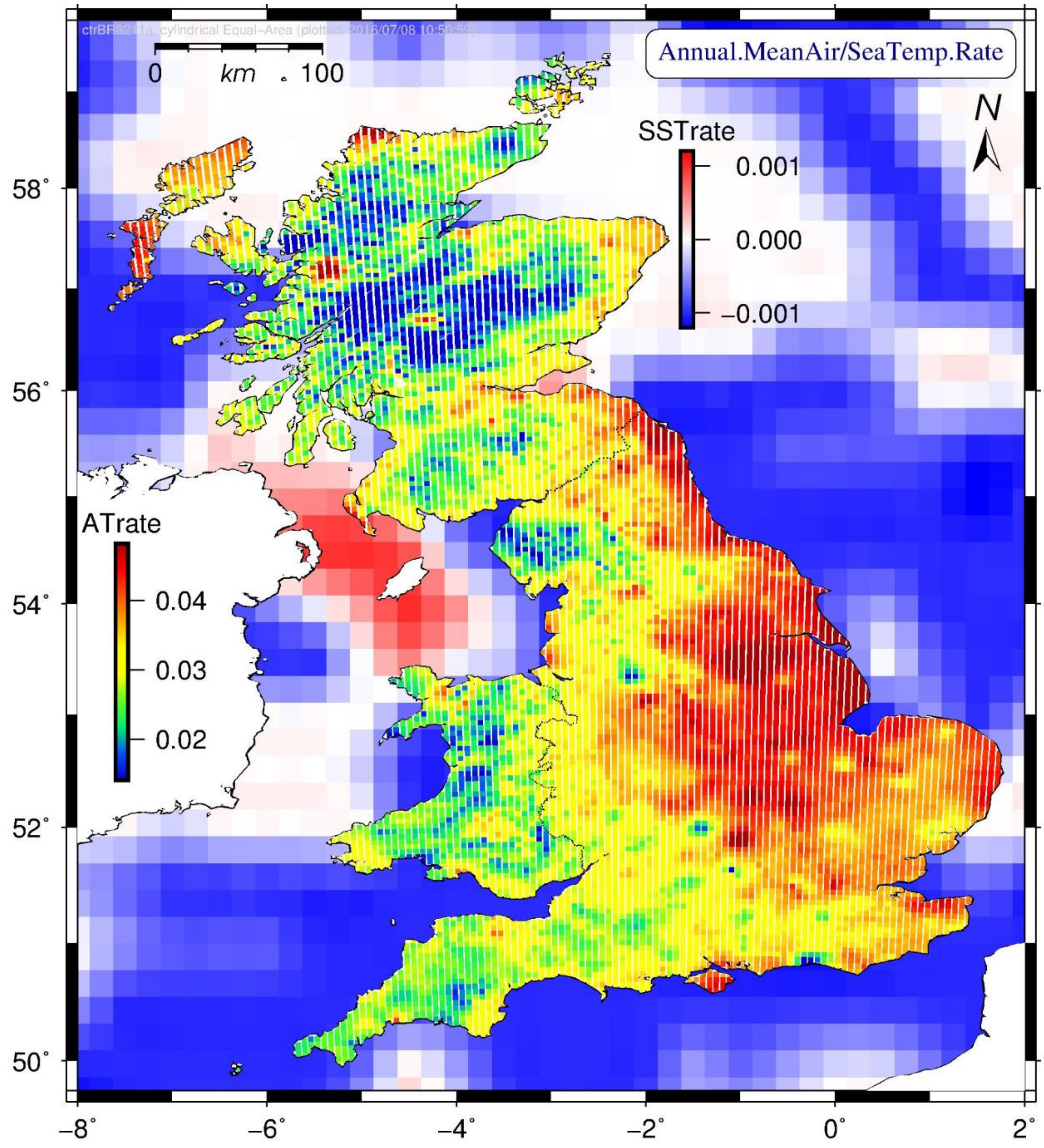

Figure A. Annual warming rates per grid cell over the studied interval (1982-2011), for Air Temperature (AT, per 5x5 km; mean 0.031 ^{o}C/yr) and Sea Surface Temperature (SST, per ¼-degree; mean: -4.45e-4 ^{o}C/yr).

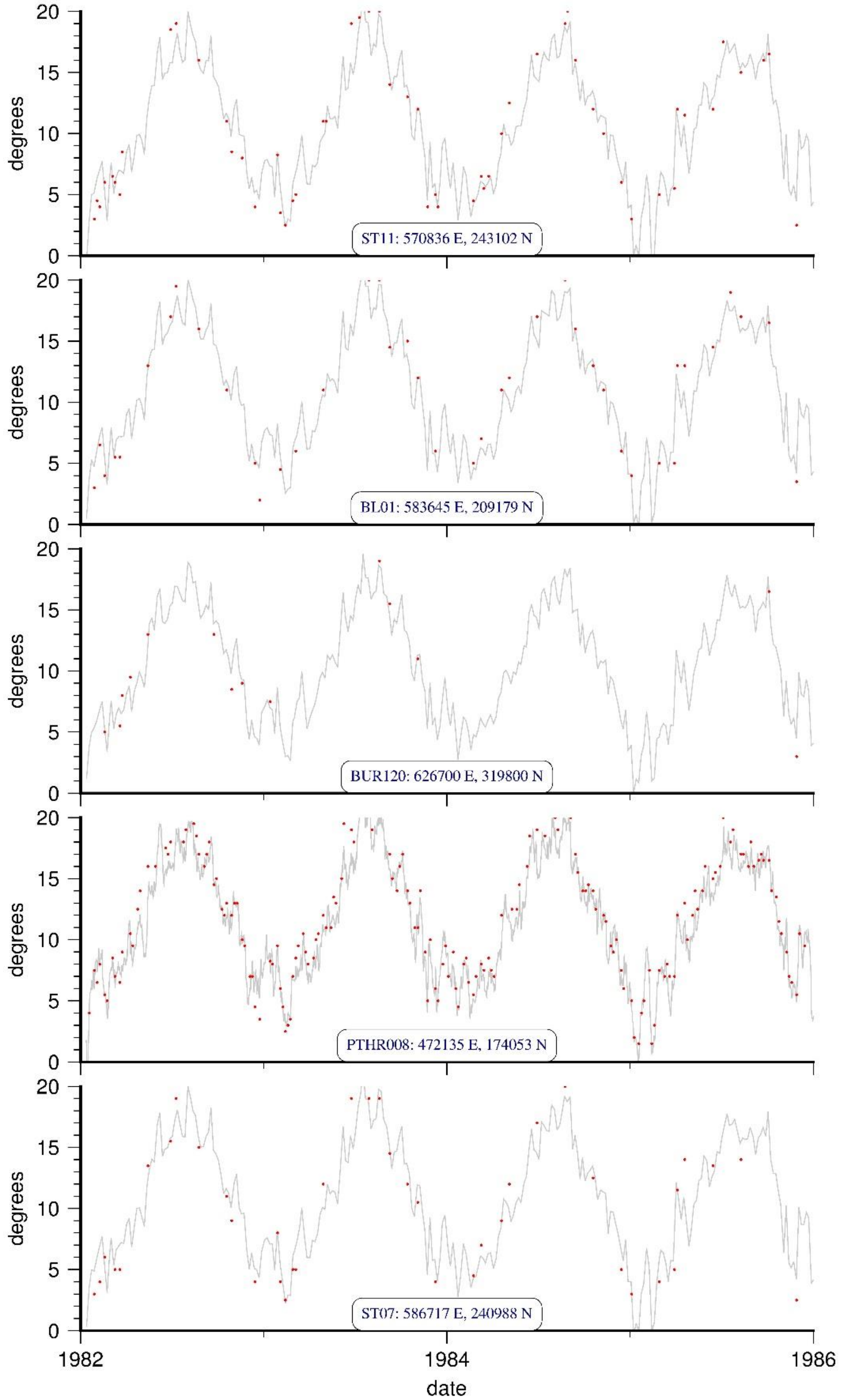

Figure B. Out-of-sample hindcasts of the candidate model (1987-2006; grey lines) and SWTA daily means (red dots) in the period 1982-1986, for several randomly picked locations with over twenty-five years of data. Temporal resolution: 5 days.

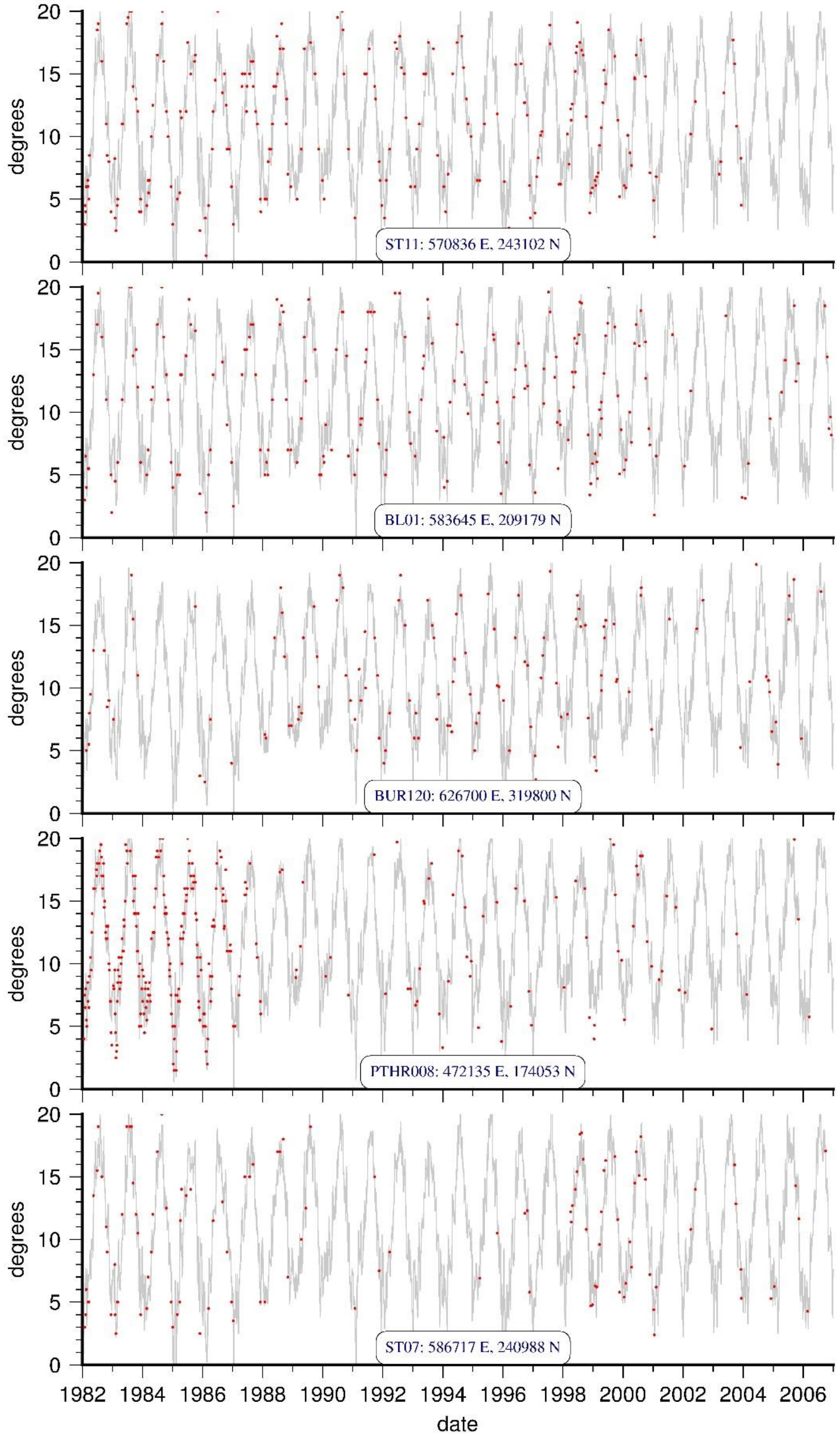

Figure C. In-sample predictions of final model (1982-2011; grey lines) and SWTA daily means (red dots) for the same locations as in the previous figure. Temporal resolution: 5 days.

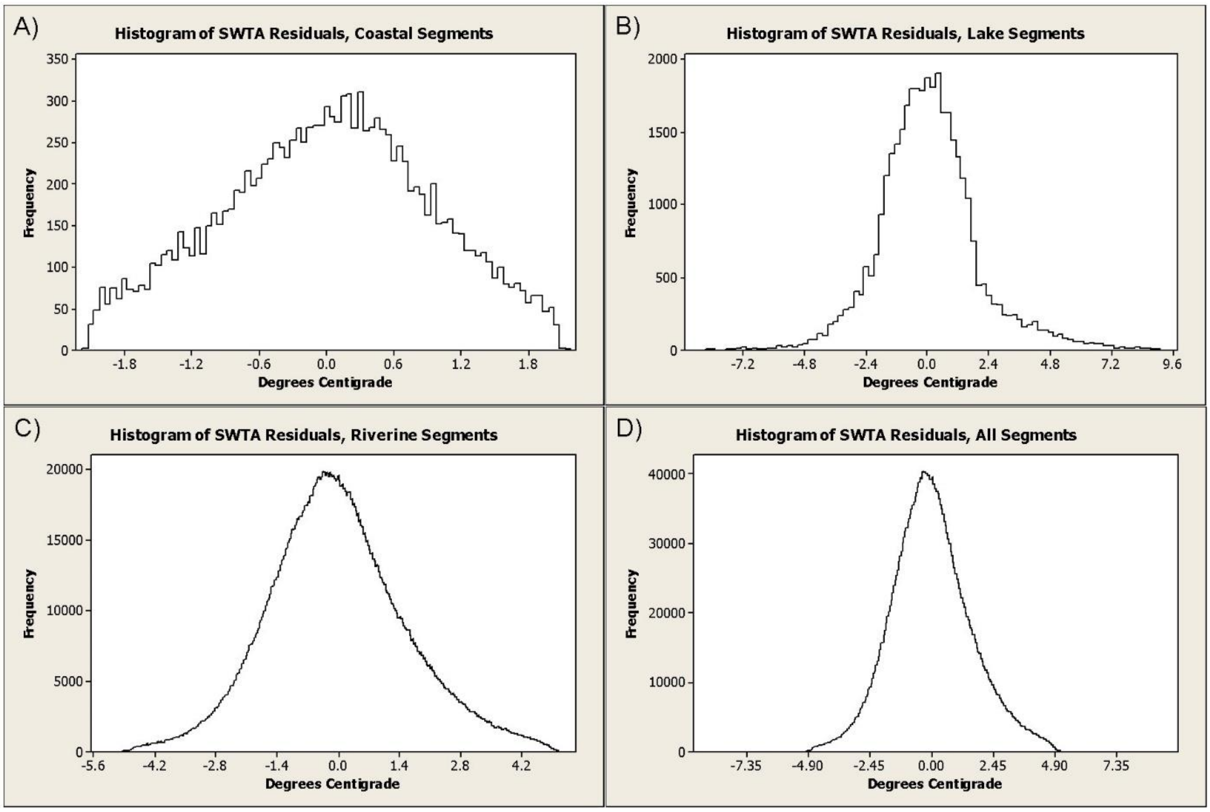

Figure D. Final model's residuals for SWTA observational daily means per segment class and overall, after outlier removal. (A) Model residuals for coastal segment-associated SWTA daily means (13,627 points, mean: 0.0003 ^{o}C, standard deviation (StDev): 0.93 ^{o}C); (B) Model residuals for lacustrine segment-associated SWTA daily means (37,138 points, mean: 0.065 ^{o}C, StDev: 1.97 ^{o}C); (C) Model residuals for riverine segment-associated SWTA daily means (2,763,342 points, mean: -0.018 ^{o}C, StDev: 1.58 ^{o}C); (D) All residuals combined (2,814,107 points, mean: -0.017 ^{o}C, StDev: 1.59 ^{o}C). See also Table 4 in main text.

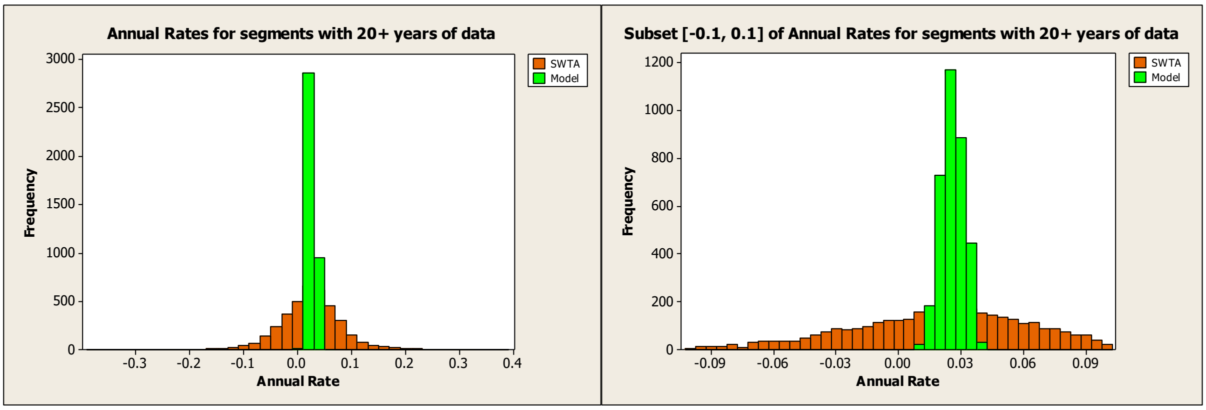

Figure E. Histograms of modelled (green) and observed (brown) annual warming rates for segments with 20+ years of SWTA measurements (daily means of raw measurements). *Left*: all 3,825 annual rates, bin width: 0.02 ^{o}C/yr. *Right*: subset range: -0.1 to +0.1/yr (3,459 annual rates), bin width: 0.005 ^{o}C/yr. The model greatly reduces the implausibly broad range of rates as derived from the (daily means of the) raw SWTA observations. A paired *t*-test for the means of the two data sets shows no significant differences (p=0.328), suggesting the model adequately captures the central tendency of observed annual warming.

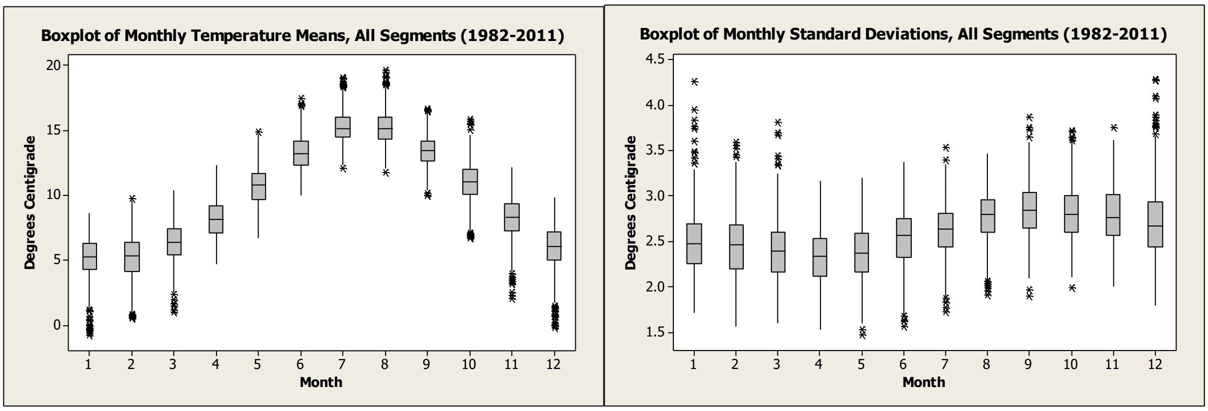

Figure F. Boxplots of monthly mean temperature (left panel) and standard deviation (right panel) over all 20,578 British river segments (1982-2011). Box marks interquartile range with midbar = median; whiskers (vertical line) mark upper (Q3 + 1.5 (Q3 – Q1)) and lower limit (Q1– 1.5 (Q3 – Q1)); stars = outliers. *Left*: July and August are the warmest months. *Right*: April (the fastest-warming month) displays least variability across Britain, whereas the winter months have most.

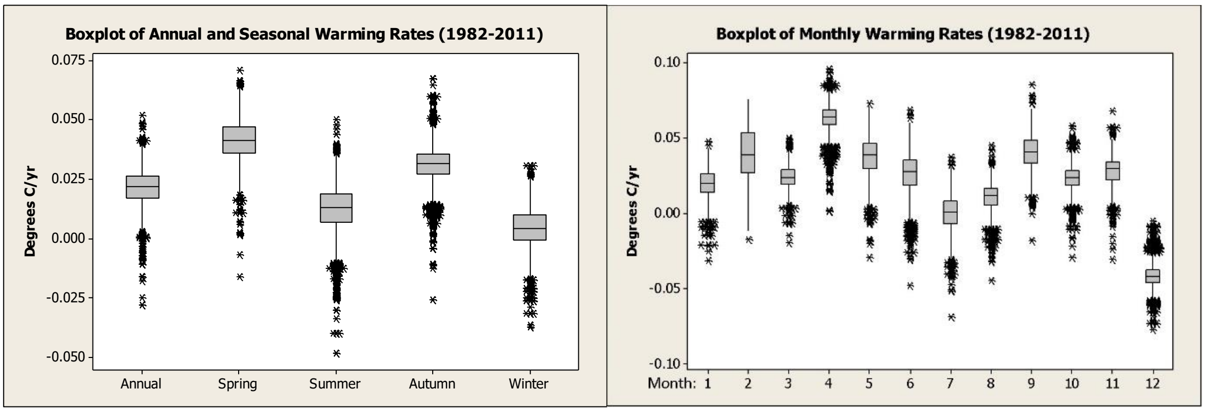

Figure G. Boxplots of annual, seasonal, and monthly warming rates over all 20,578 British river segments (1982-2011). Box marks interquartile range with midbar = median; whiskers (vertical line) mark upper (Q3 + 1.5 (Q3 – Q1)) and lower limit (Q1– 1.5 (Q3 – Q1)); stars = outliers. *Left*: Spring and Autumn are warming fastest. *Right*: April is the fastest-warming month; December is the only cooling month.

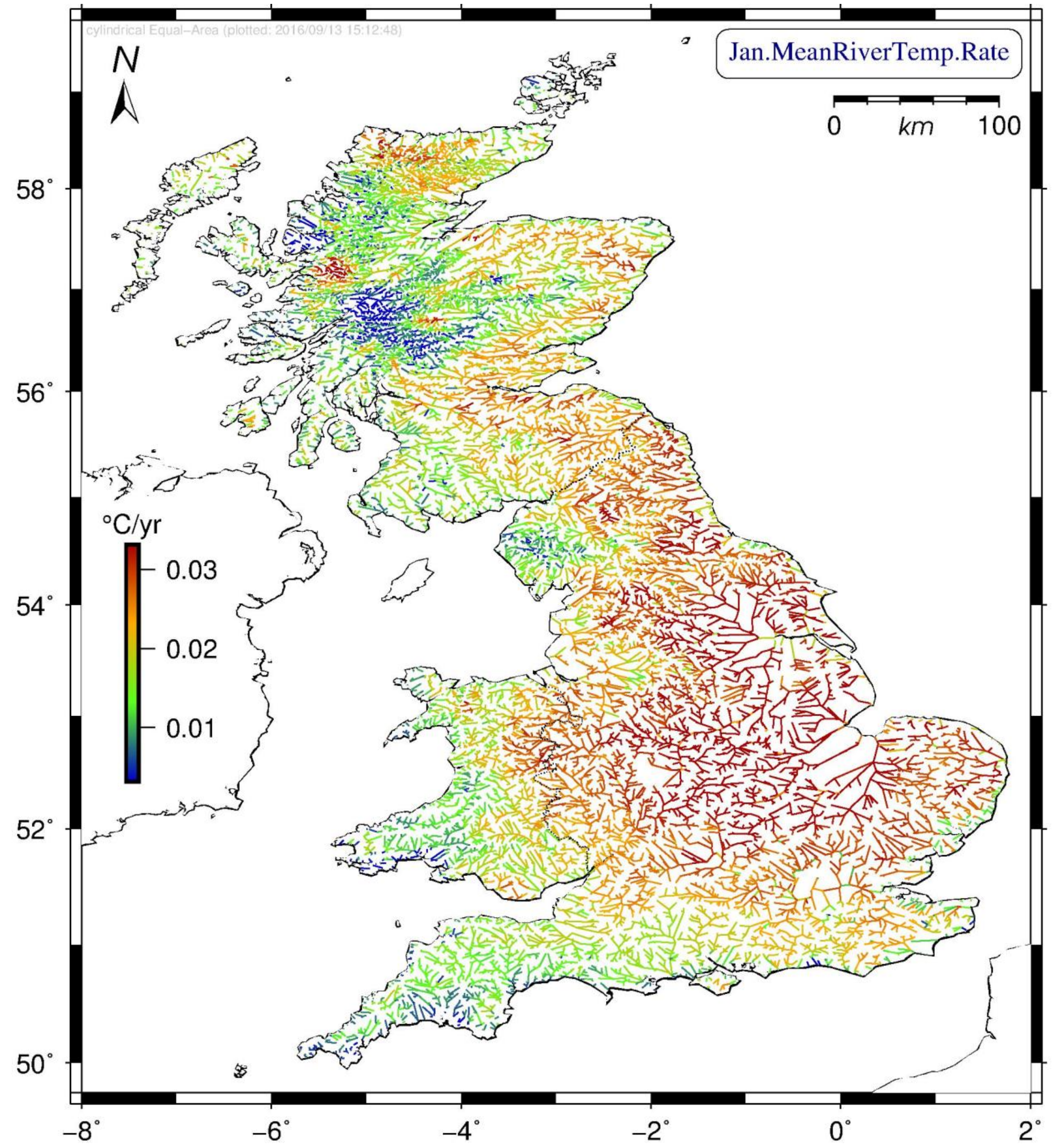

Figure H. Modelled annual warming rates (^{0}C /year) for British river segments in the month of January (1982-2011). Scottish model results extrapolate English and Welsh data.

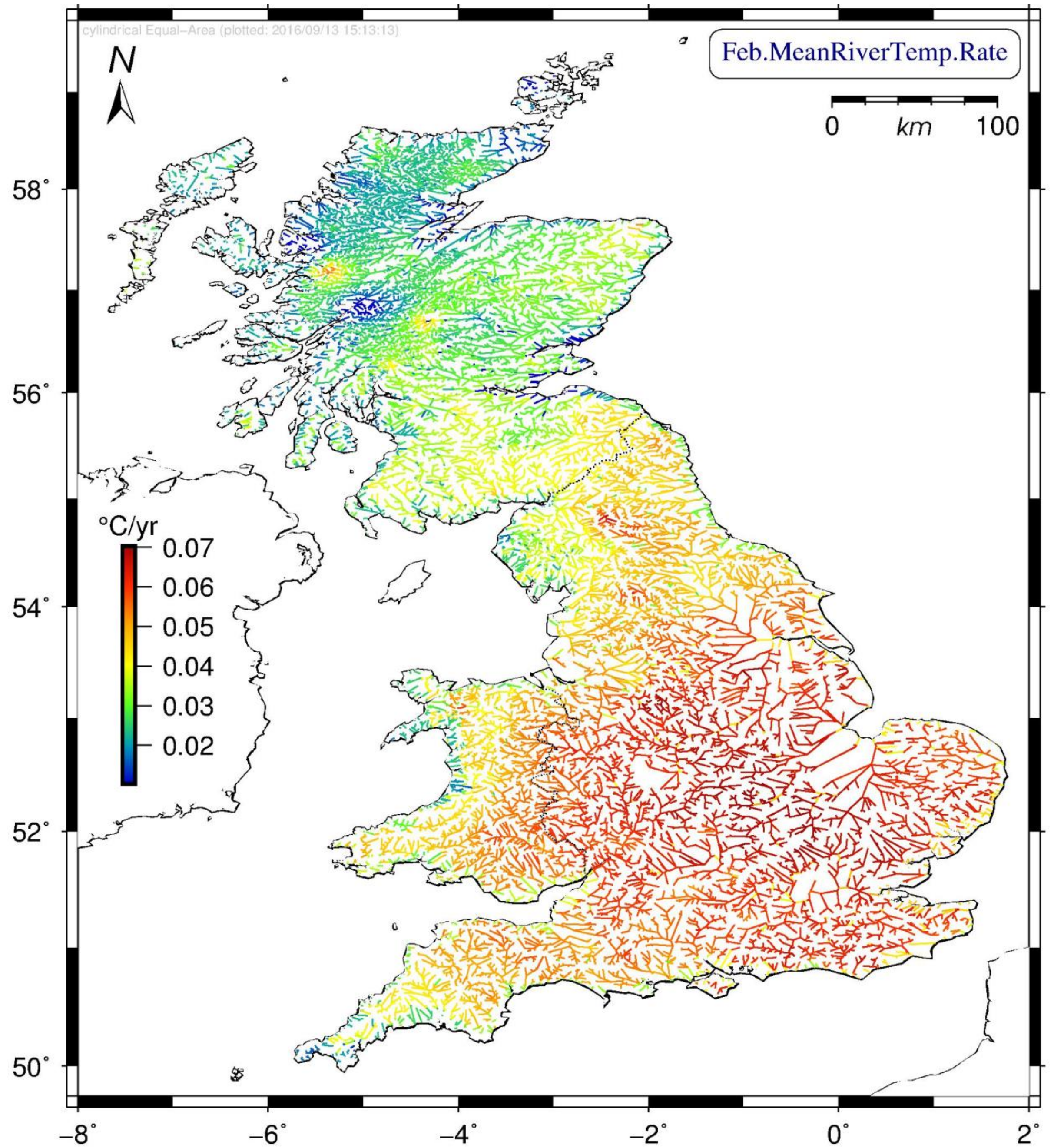

Figure I. Modelled annual warming rates (^{o}C /year) for British river segments in the month of February (1982-2011). Scottish model results extrapolate English and Welsh data.

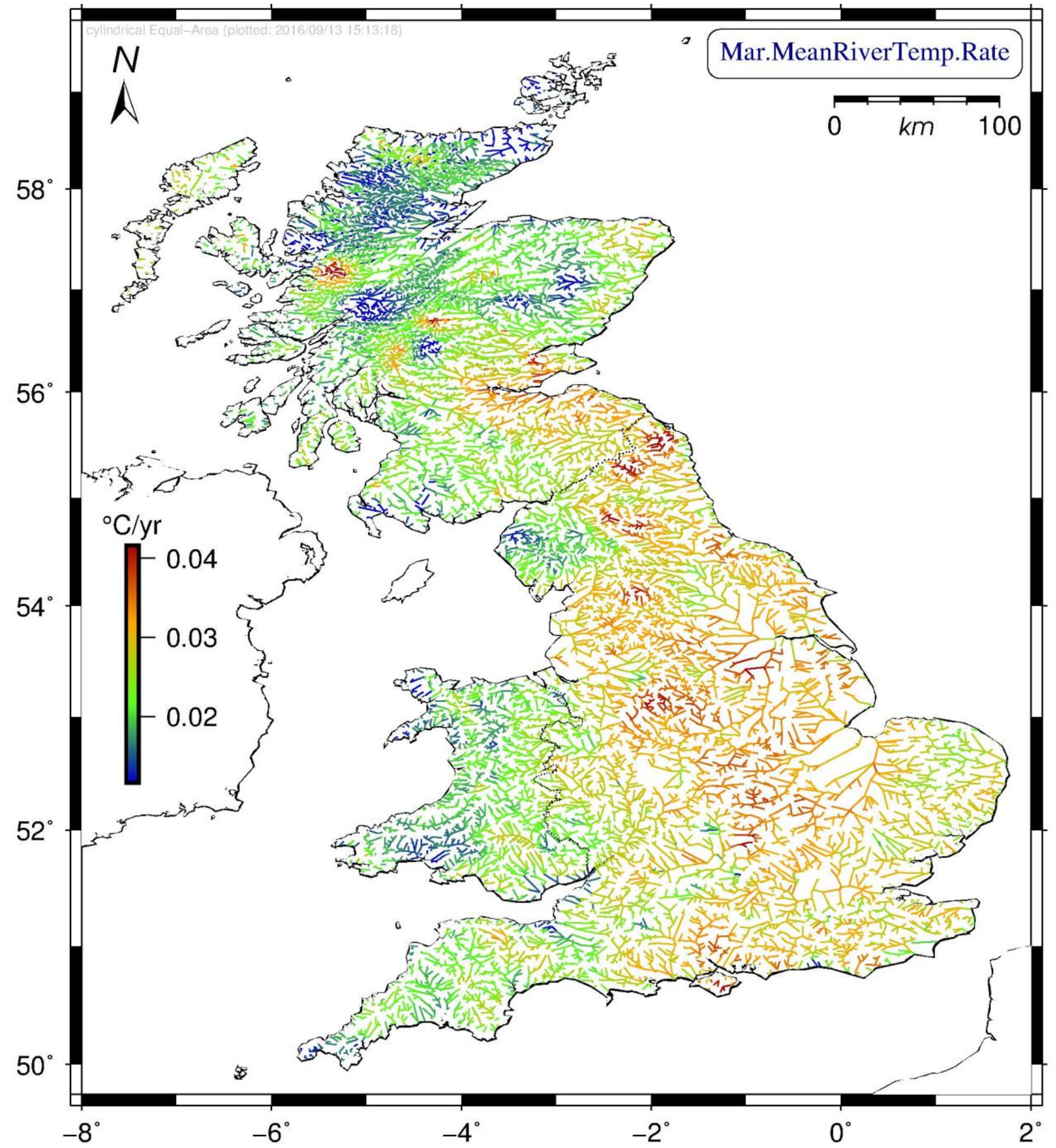

Figure J. Modelled annual warming rates (^{0}C /year) for British river segments in the month of March (1982-2011). Scottish model results extrapolate English and Welsh data.

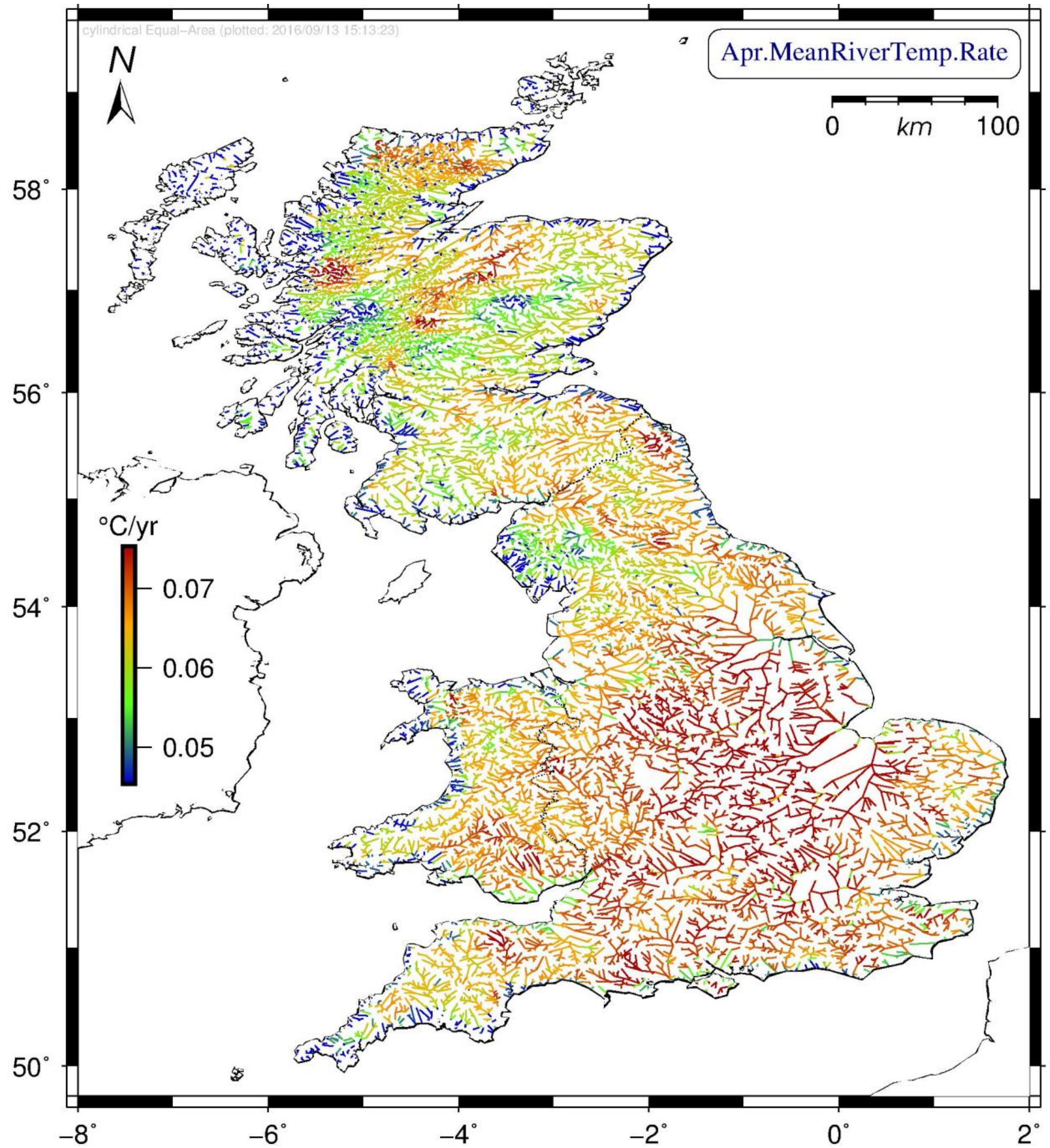

Figure K. Modelled annual warming rates (^{0}C /year) for British river segments in the month of April (1982-2011). Scottish model results extrapolate English and Welsh data.

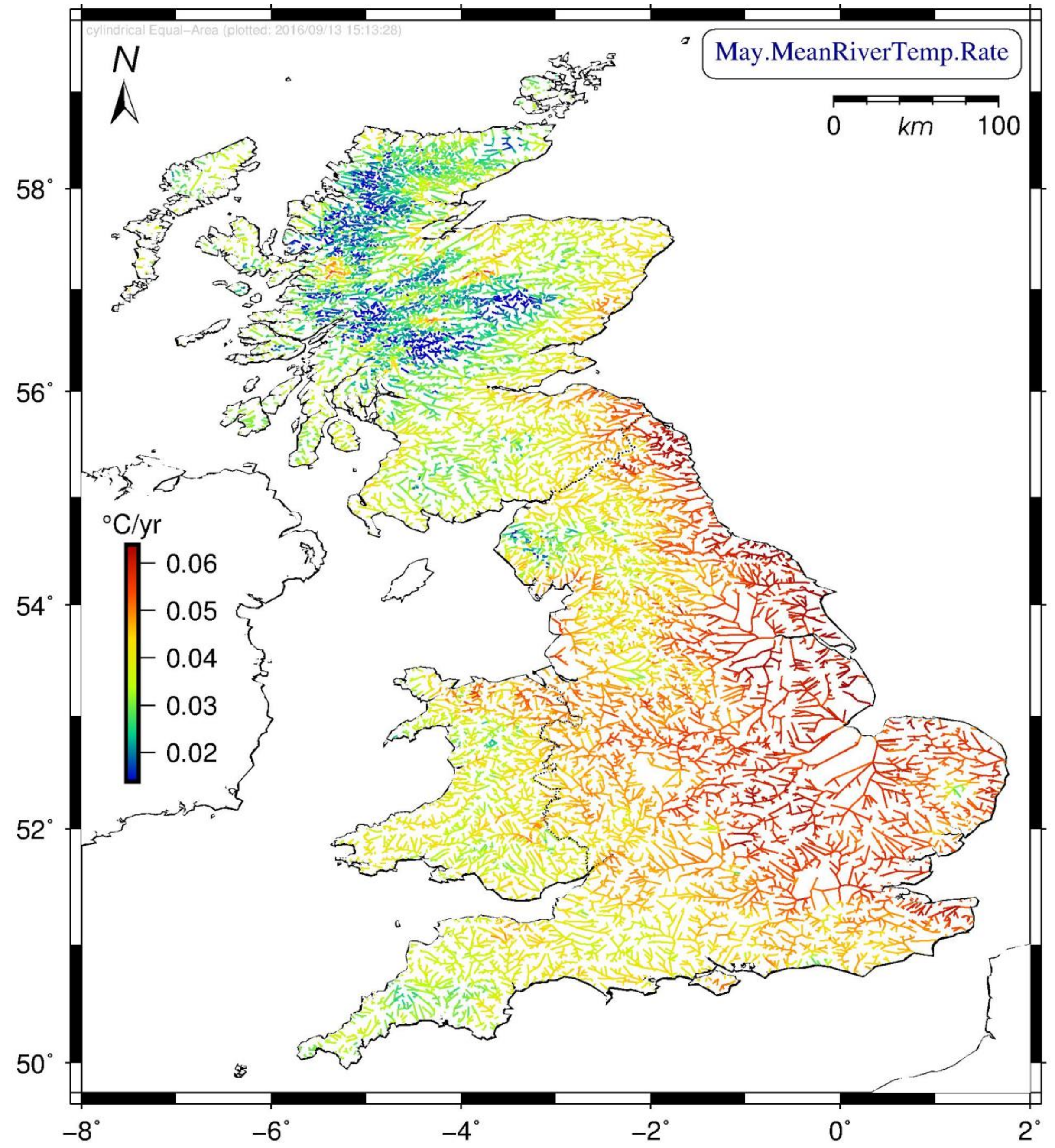

Figure L. Modelled annual warming rates (^{o}C /year) for British river segments in the month of May (1982-2011). Scottish model results extrapolate English and Welsh data.

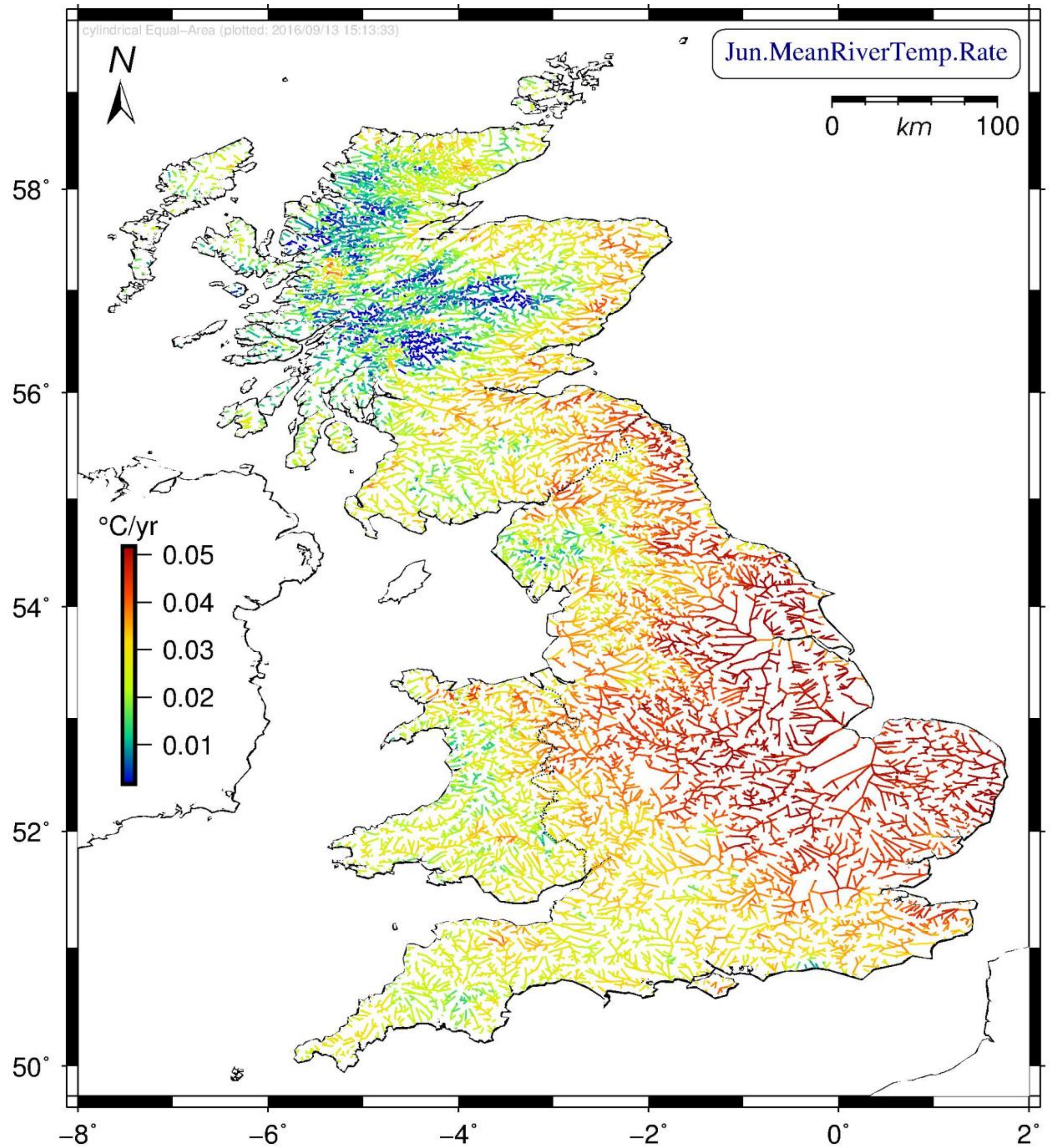

Figure M. Modelled annual warming rates (°C /year) for British river segments in the month of June (1982-2011). Scottish model results extrapolate English and Welsh data.

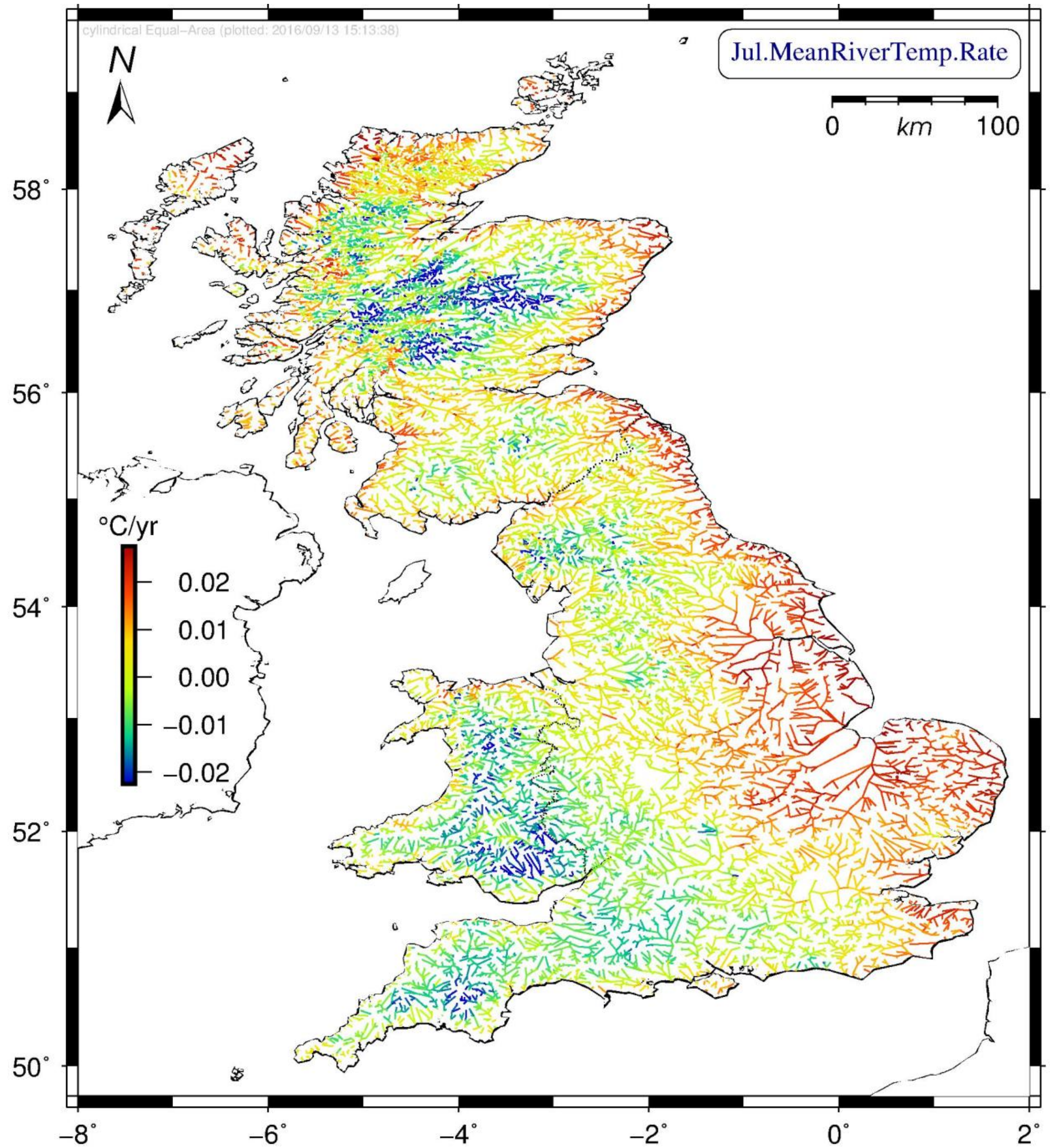

Figure N. Modelled annual warming rates (°C /year) for British river segments in the month of July (1982-2011). Scottish model results extrapolate English and Welsh data.

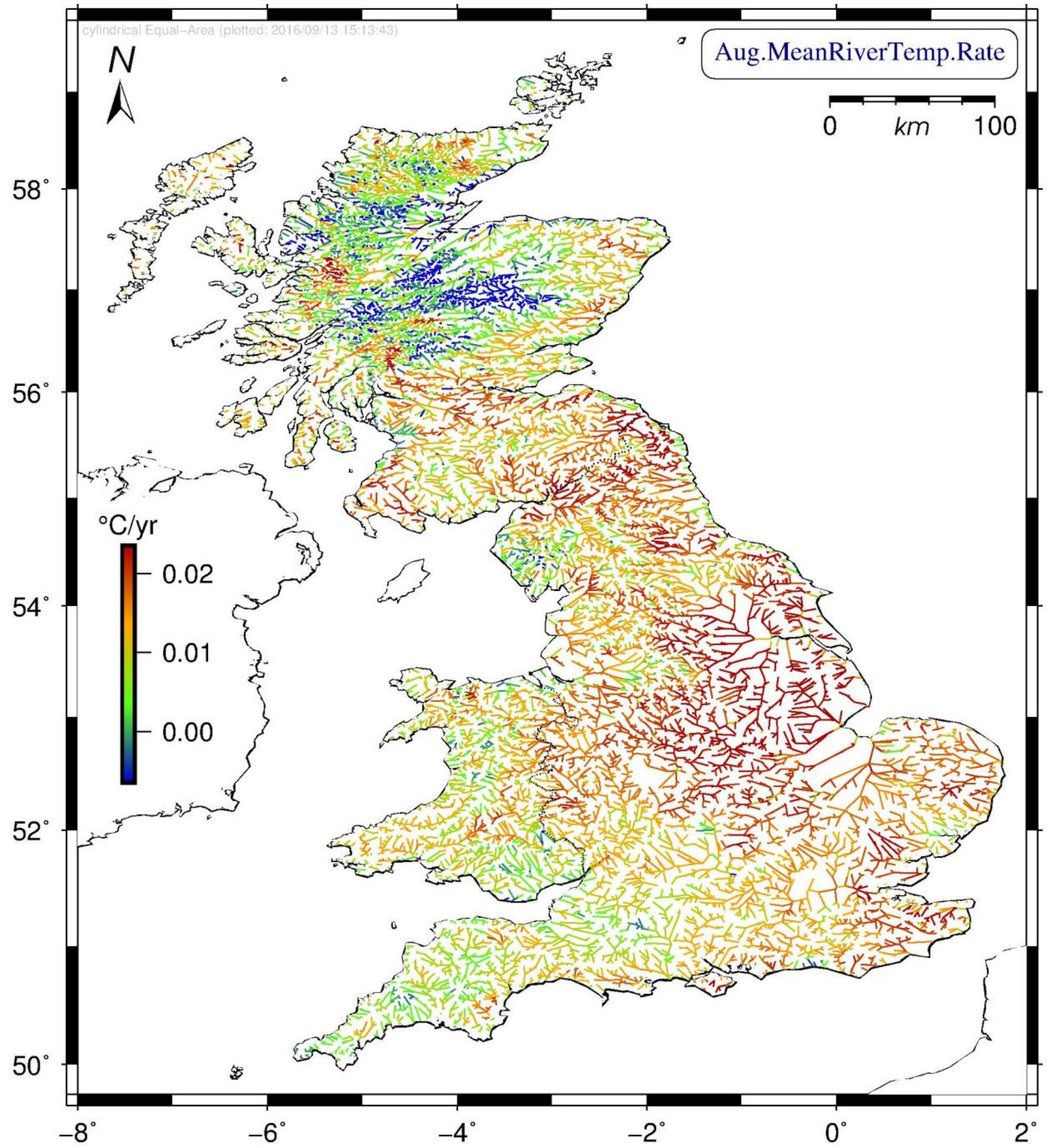

Figure O. Modelled annual warming rates (°C /year) for British river segments in the month of August (1982-2011). Scottish model results extrapolate English and Welsh data.

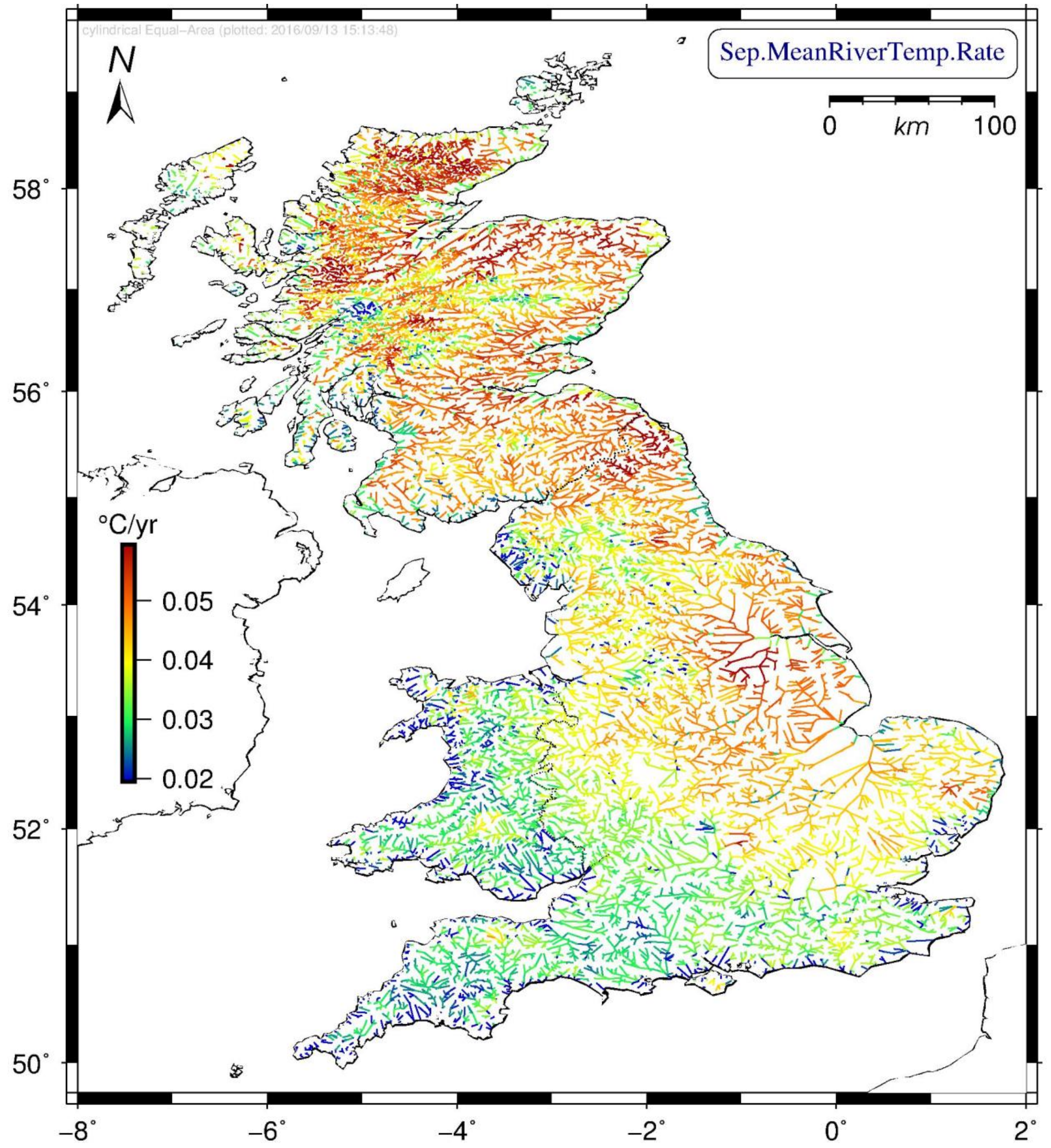

Figure P. Modelled annual warming rates (ºC /year) for British river segments in the month of September (1982-2011). Scottish model results extrapolate English and Welsh data.

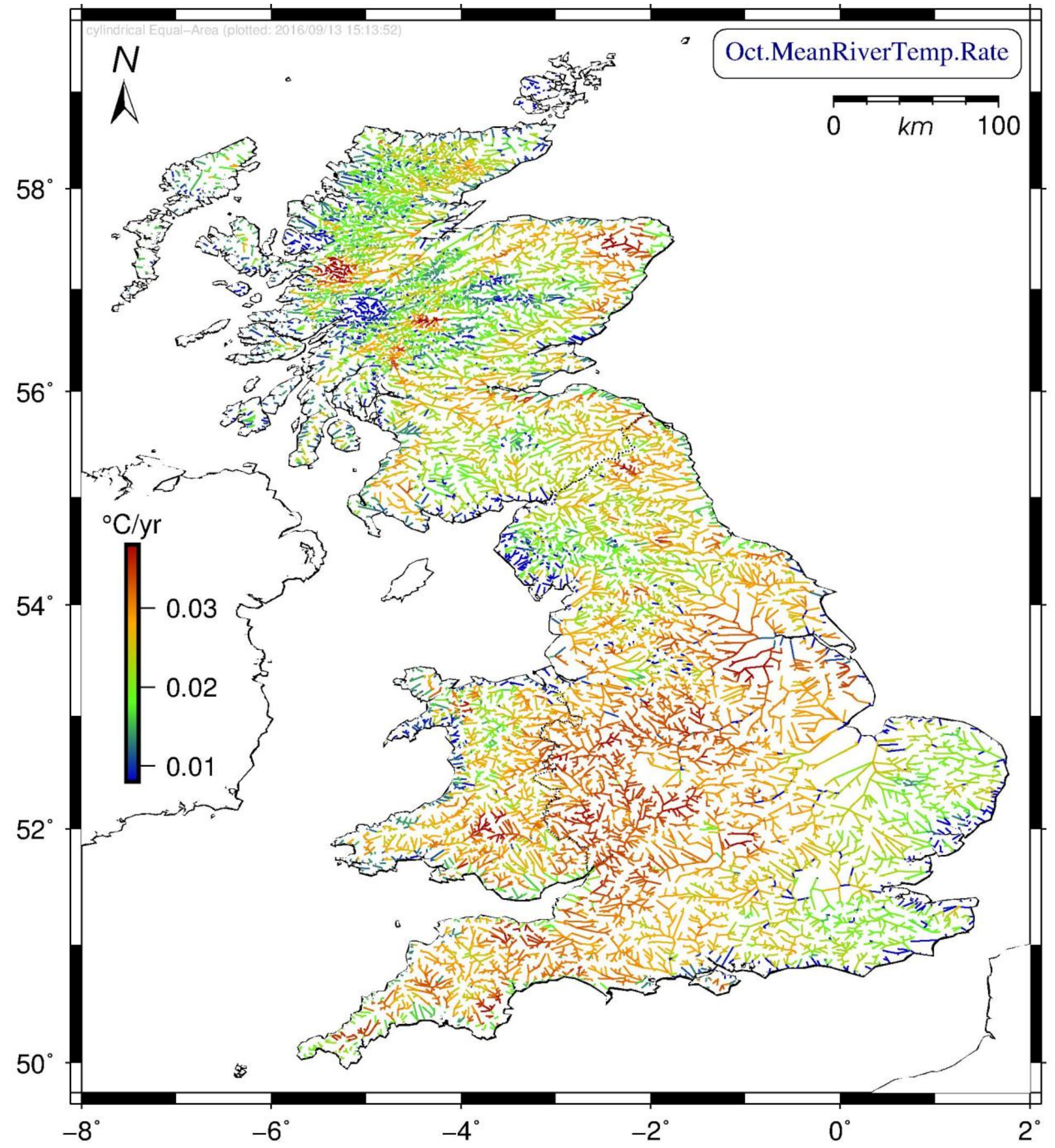

Figure Q. Modelled annual warming rates (^{0}C /year) for British river segments in the month of October (1982-2011). Scottish model results extrapolate English and Welsh data.

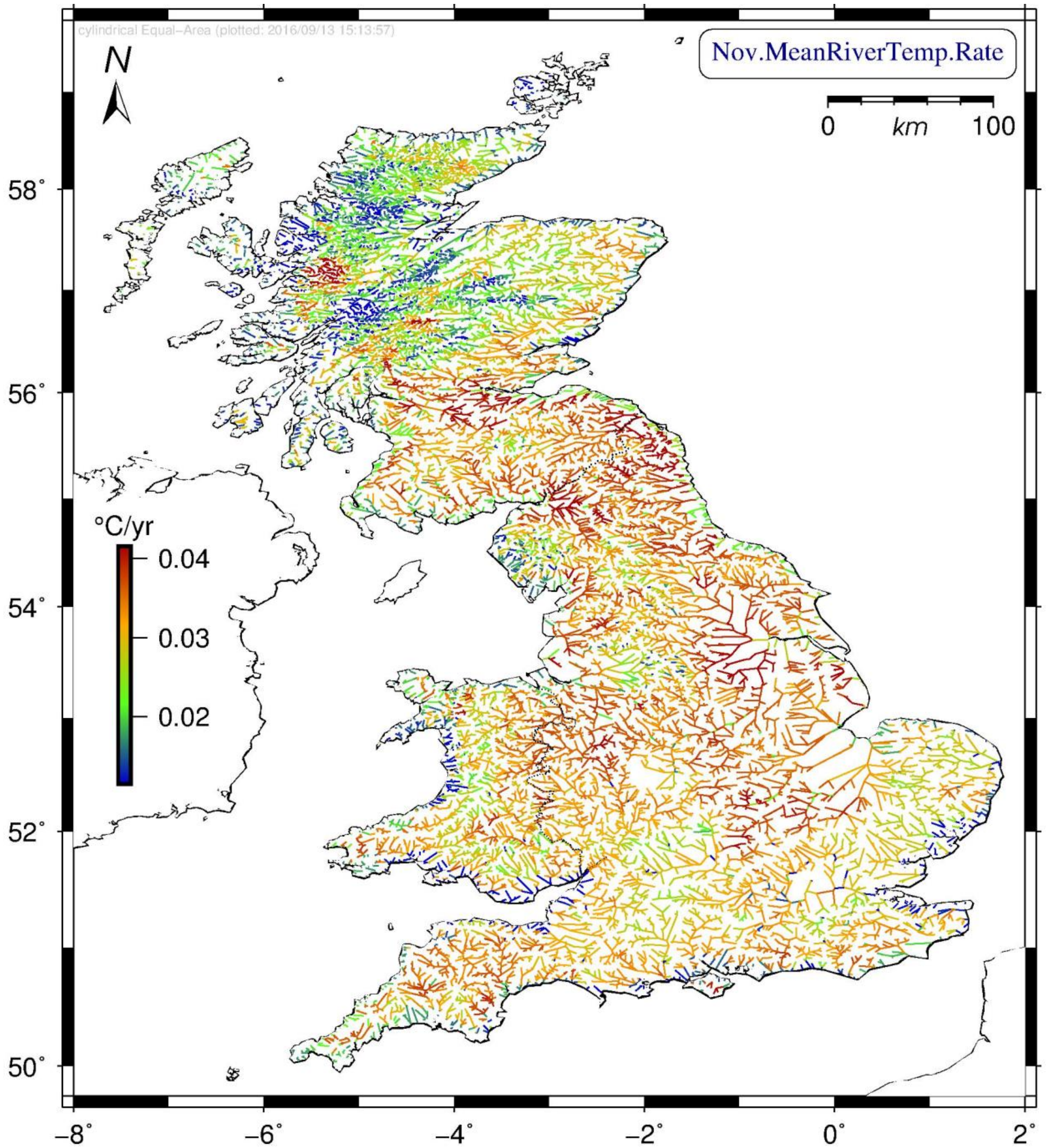

Figure R. Modelled annual warming rates (⁰C /year) for British river segments in the month of November (1982-2011). Scottish model results extrapolate English and Welsh data.

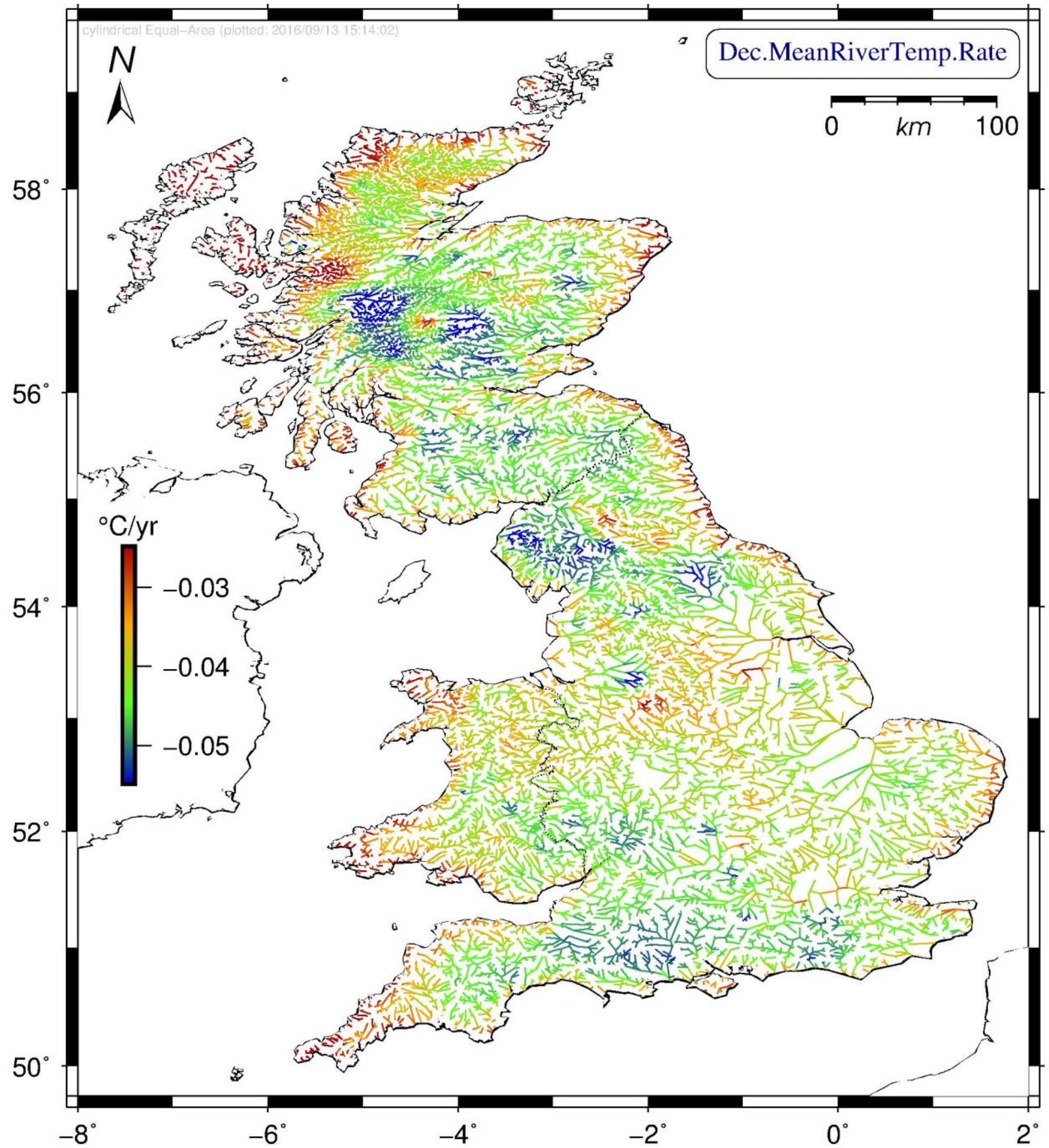

Figure S. Modelled annual warming rates (⁰C /year) for British river segments in the month of December (1982-2011). Scottish model results extrapolate English and Welsh data.

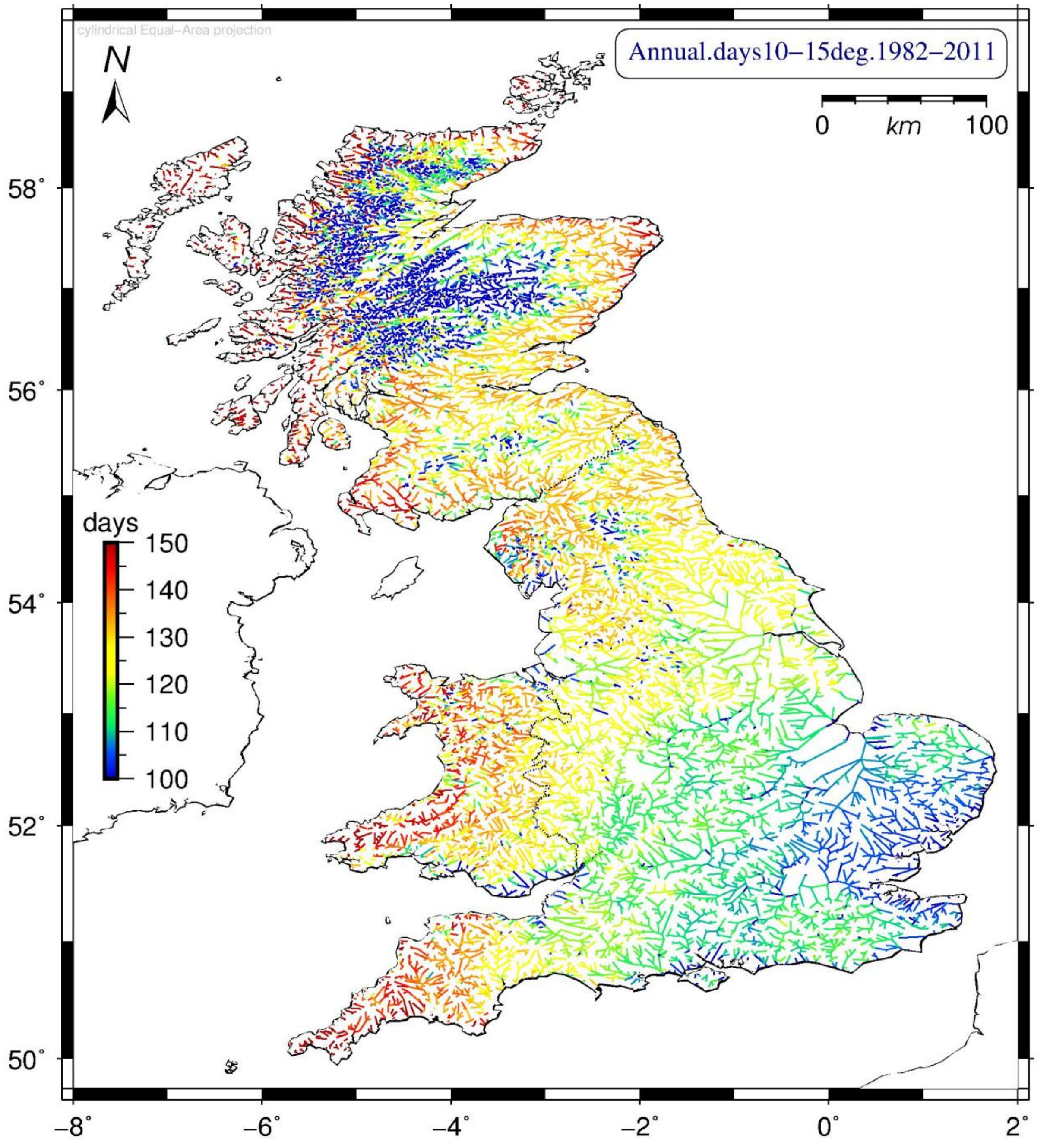

Figure T. Modelled mean number of days per year river segments spent in the temperature bracket from 10 to 15 ^{o}C (the most high-risk for large outbreaks of fish diseases) over the studied interval of 1982-2011. The Scottish Highlands and southeast England were at lowest risk. Scottish model results extrapolate English and Welsh data.

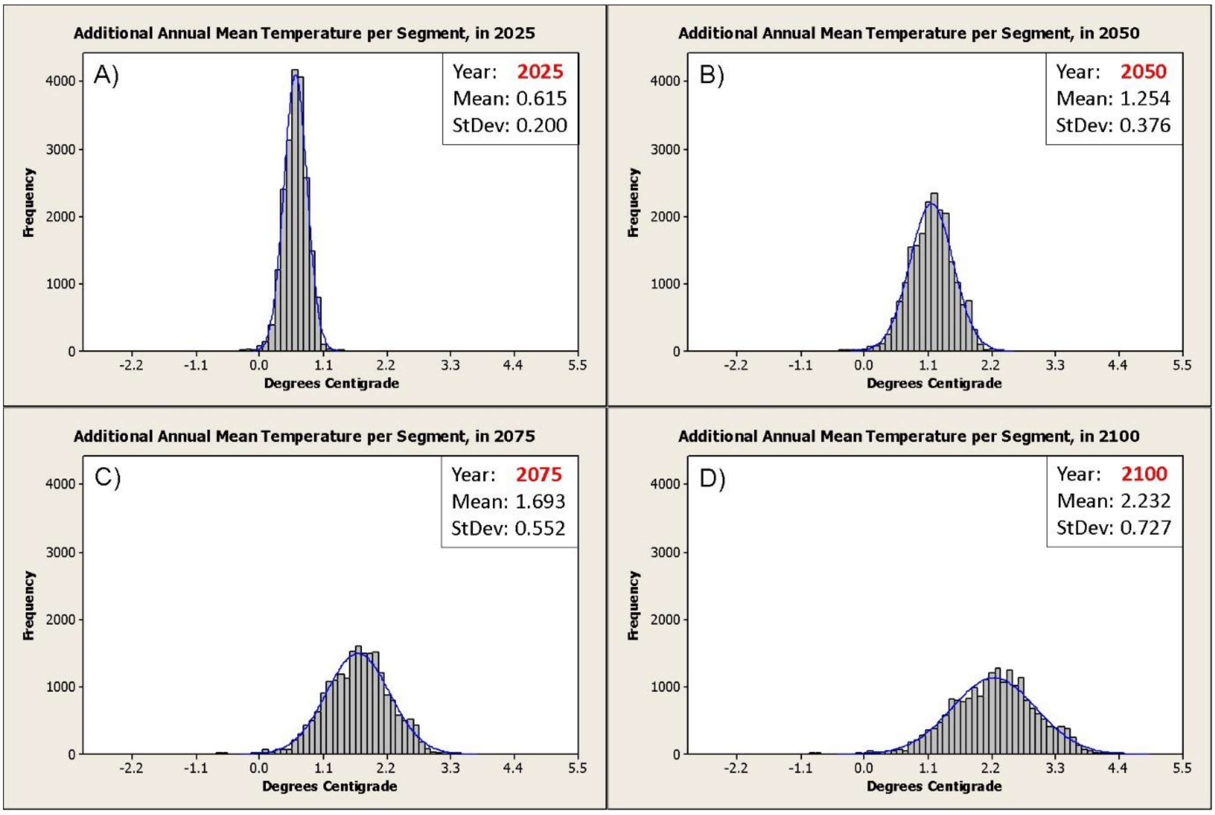

Figure U. Histograms of modelled additional warming in ^{o}C per river segment for epochs 2025, 2050, 2075, and 2100 (A-D), relative to mean water temperature per segment over the period 1982-2011. Mean warming increases from +0.62 ^{o}C in 2025 to +2.23 ^{o}C in 2100, with an approximate Gaussian distribution around it (blue bell curve), which by 2100 ranges from below zero to over +4.5 ^{o}C of warming for individual segments; the +/-2 sigma range then spans +0.81 to +3.66 ^{o}C.
